# The adhesion GPCR ADGRV1 controls glutamate homeostasis in hippocampal astrocytes supporting neurons

**DOI:** 10.1186/s40478-026-02282-2

**Published:** 2026-04-19

**Authors:** Baran E. Güler, Mark Zorin, Joshua Linnert, Kerstin Nagel-Wolfrum, Uwe Wolfrum

**Affiliations:** 1 Institute of Molecular Physiology, Molecular Cell Biology, Johannes Gutenberg University Mainz, Hanns-Dieter-Hüsch-Weg 17, 55099 Mainz, Germany; 2 Institute of Developmental Biology and Neurobiology, Johannes Gutenberg University Mainz, 55099 Mainz, Germany; 3 Institute for Quantitative and Computational Biosciences (IQCB), Johannes Gutenberg University Mainz, 55099 Mainz, Germany; 4 Institute of Human Genetics, Heidelberg University, Im Neuenheimer Feld 366, 69120 Heidelberg, Germany

**Keywords:** Adhesion GPCR, VLGR1, Epilepsy, Usher syndrome, Astroglia, Glutamate-glutamine cycle, Glutamate metabolism

## Abstract

ADGRV1 is the largest member of adhesion G protein-coupled receptor (aGPCR) family. In the cell, aGPCRs serve in two major functions, namely in cell adhesion and signal transduction. Mutations in *ADGRV1* were linked not only to Usher syndrome (USH), which causes deaf-blindness, but recently, also to epilepsy. While the USH defects are attributed to the loss of fiber links between membranes formed by the extracellular domain of ADGRV1, the pathomechanisms leading to epilepsy remain elusive to date. Here, we study the specific functions of ADGRV1 in astrocytes, where it is most highly expressed in the nervous system. Affinity proteomics demonstrated the interaction of ADGRV1 with proteins enriched in astrocytes. Different transcriptomes of USH2C patient-derived cells and Adgrv1-deficient mouse hippocampi compared to controls indicated dysregulation of cellular processes important in astrocyte function. Cell counts and morphometric analysis revealed reduced numbers and altered morphology of astrocytes in the hippocampus of Adgrv1-mutant mice. Monitoring the glutamate uptake in colorimetric assay and by live cell imaging of a genetic glutamate reporter consistently showed that glutamate uptake from the extracellular environment is significantly reduced in Adgrv1-deficent astrocytes. Expression analyses of key enzymes of the glutamate glutamine cycle and the glutamate metabolism indicated imbalanced glutamate homeostasis in Adgrv1-deficient astrocytes. Finally, we provide evidence that the supportive function of astrocytes in neuronal development also relies on ADGRV1 expression in astrocytes. Our data collectively provide first insights into the molecular pathophysiology associated with ADGRV1 defects in the brain, which may relate to the development of epilepsy associated with mutations in *ADGRV1*.

**Graphical Abstract:**

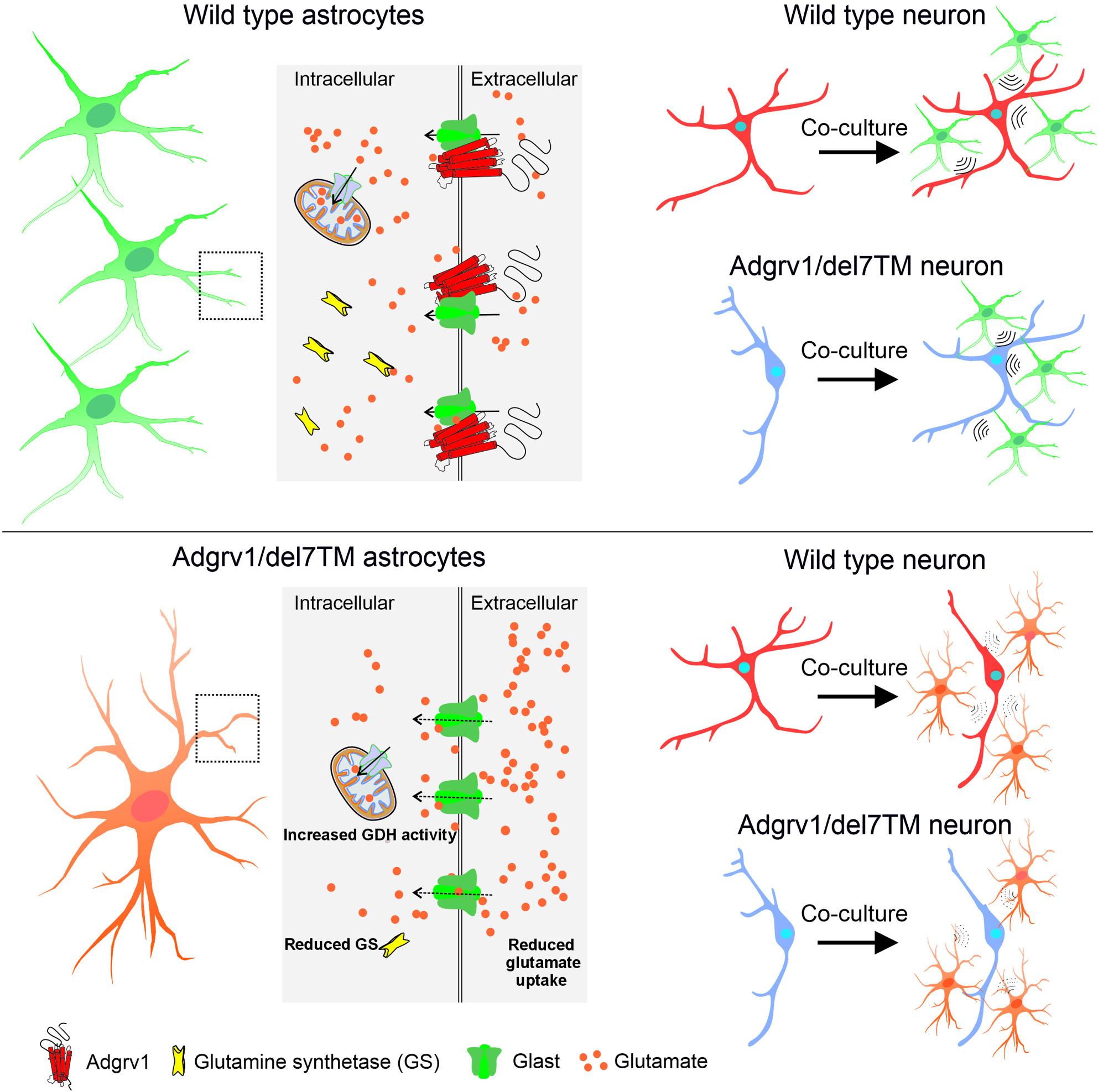

**Supplementary Information:**

The online version contains supplementary material available at 10.1186/s40478-026-02282-2.

## Main Points

- Deficiency in ADGRV1 results in depletion and morphologic alterations of hippocampal astrocytes.
- Omics data demonstrates the association of ADGRV1 with proteins and processes concentrated in astrocytes.
- ADGRV1 balances glutamate homeostasis by controlling glutamate uptake, glutamate-glutamine cycle, and glutamate metabolism in astrocytes.
- ADGRV1 in astrocytes is vital for neuron morphogenesis.
- Unbalanced glutamate homeostasis in astrocytes may underlie the development of epilepsy associated with mutations in *ADGRV1*.

**Supplementary Information:**

The online version contains supplementary material available at 10.1186/s40478-026-02282-2.

## Introduction

ADGRV1 (previously known as GPR98, MASS1, or VLGR1) with a molecular weight of approximately 700 kDa is the largest member of the adhesion G protein-coupled receptor (aGPCR) family consisting of 33 other members [1]. Adhesion GPCRs are structurally chimeric containing signature domains of seven transmembrane receptors and adhesion proteins [2]. This repertoire makes them suitable to participate in cell adhesion and signaling through G protein coupled pathways [3]. Like for other aGPCRs, ADGRV1 can be autoproteolytically processed at its GAIN domain and act as a metabotropic mechanoreceptor [4]. Mechanical stimulation of ADGRV1 could trigger its autoproteolytic cleavage, and released the tethered “Stachel” agonist, inducing the switch from Gαs- to Gαi-mediated signaling [3]. In cells and tissue ADGRV1 plays a role as a component of fibrous membrane-links, membrane contacts to the extracellular matrix, namely focal adhesions and in organelle contact sites such as the mitochondria-associated ER membranes (MAMs) [5–8]. There, ADGRV1 was related to the control of cell spreading and migration and the maintenance of Ca^2+^-homeostasis.

In humans, pathogenic variants in *ADGRV1* cause USH2C, one of the 11 subtypes of the human Usher syndrome, which is overall the most common form of hereditary deaf blindness [9]. In the sensory cells of the eye and ear which are affected by USH, there is evidence that the loss of fibrous links between adjacent membranes formed by the long extracellular domain of ADGRV1 leads to the dysfunction underlying USH2C [5, 10]. Recently rare variants of *ADGRV1* have been associated with other diseases affecting the inner ear namely with sporadic vestibular Schwannoma and Meniere disease [11, 12]. However, in recent years pathogenic variants in *ADGRV1* have been linked in particular to various forms of epilepsy [13–18]. In cohorts of patients with confirmed epilepsy, the rate of *ADGRV1* pathogenic variants was determined to be nearly 3% [16]. Although most of these epilepsy cases related to *ADGRV1* haploinsufficiency have good prognoses, a recent case report of a heterozygous missense mutation of *ADGRV1* (c.5785G > T) in a patient resulted in hippocampal sclerosis characterized by neuronal cell loss and sudden unexpected death [19]. It is generally accepted that the epileptic seizures which are characteristic for epilepsy are induced by excessive and abnormal neuronal activity in the brain [20]. However, the molecular mechanisms leading to the different forms of epilepsy remain elusive to date [21, 22]. Also, to date, nothing is known about the molecular and cellular pathomechanisms that lead to the epileptic brain conditions caused by defects in *ADGRV1*.

Although ADGRV1 is expressed almost ubiquitously in many tissues, its expression is strongest in the developing and mature nervous system [23]. Of all the cell types in the brain, astrocytes express ADGRV1 the most strongly (https://www.proteinatlas.org/). Astrocytes are glia cells and one of the most abundant cells in the central nervous system (CNS). They have many roles including regulation of extracellular ion concentration, control of energy metabolism, glutamate clearance, neuroprotection, synaptogenesis, and establishment of the blood-brain barrier [24, 25]. The branching of astrocytes promotes extensive contacts with neighboring cells, in particular neurons and specific association with synapses [26]. In tripartite synapses, astrocytes wrap around the pre- and post-synapses of neurons and regulate the crosstalk between synapses by balancing the ion and glutamate homeostasis [27–29]. Glutamate uptake by astrocytes clears the glutamate spillover from the synaptic cleft and is part of the glutamate-glutamine cycle between astrocytes and neurons preventing glutamate toxification of the neural tissue [24, 30]. However, the uptaken glutamate can be also metabolized in the astrocytes for energy production in the mitochondrial tricarboxylic acid cycle (TCA) [31]. The dysregulation of both the glutamate-glutamine cycle and the glutamate metabolism in astrocytes can cause an imbalance in brain glutamate homeostasis and lead to hyperexcitability of neurons as seen in epilepsy [32]. Loss of astrocyte functions or defects in their migratory capacity during development, or postnatal dysfunctions of astrocytes, have been associated not only with neurodegenerative diseases, such as Alzheimer´s and Parkinson’s disease, but also with the development of various forms of epilepsy [33–35].

Although ADGRV1 is abundantly expressed in astrocytes, little is known about its specific function in astrocytes of the CNS. In previous studies on the function of ADGRV1, we employed primary astrocytes derived from murine brains as cellular models with no specific focus on the potentially epilepsy-relevant functions in the brain [4, 7, 8]. Our previous studies have collectively revealed insights into a variety of roles of ADGRV1 in cell functions such as cell adhesion, cell migration, mechanosensation, and the regulation of the Ca^2+^-homeostasis at MAMs [4, 7, 8].

Our present study focuses on elucidating and understanding the specific functions of ADGRV1 in astrocytes by omics, immunohisto- and cytochemistry, biochemistry and a spectrum of cell physiology assays utilizing Adgrv1-deficient mice and human patient cells as models. We show that astrocyte morphology is altered, and their number is significantly reduced especially in the CA1 region of the hippocampus of the Adgrv1-deficient mouse brain. Omics data support the association of ADGRV1 with proteins and processes specifically enriched in astrocytes. Our data also indicate an imbalance in glutamate homeostasis in hippocampal Adgrv1-deficient astrocytes resulting in a reduced glutamate uptake from the extracellular environment as well as an alteration in the glutamate glutamine cycle and glutamate metabolism. Our work in primary cultures of astrocytes and neurons suggests that ADGRV1 is also important for the proper development of neurons and showed that ADGRV1 substantially contributes to the beneficial support of astrocytes for neurons. Collectively, our data provide first insights into the molecular pathophysiology associated with *ADGRV1* defects in the brain, which may be linked to the development of epilepsy associated with mutations in the *ADGRV1* gene.

## Materials and methods

### Animals

Experiments were conducted in accordance with the guidelines set forth by the Association for Research in Vision and Ophthalmology. Mice were housed in a controlled environment with a 12/12-hour light/dark cycle with unrestricted access to food and water. Adgrv1/del7TM mice, susceptible to audiogenic seizures, have been generated with insertion of 101 bp in exon 82 of Adgrv1 with a vector containing a STOP codon which leads to deletion of 7TM domains [10]. The mice were bred on a C57BL/6 background. Previous auditory brainstem response (ABR) measurements revealed that the Adgrv1/del7TM mutant mice are profoundly deaf by 3 weeks of age [36]. Additional electroretinogram (ERG) analysis did not show significant differences in vision function between wild types and Adgrv1/del7TM mutant mice at 6 and 15 months of age except for light-adapted cone-only responses which were significantly different in 15-month-old mutants. The use of mice for research purposes was granted approval by the District Administration Mainz-Bingen under reference number 41a/177-5865-§ 11 ZVTE on April 30, 2014.

### Antibodies

The following antibodies were used: rabbit anti-Gfap (DAKO agilent, ZO334), rabbit anti-Sox9 (Abcam, ab185966), guinea pig anti-Map2 (Sysy, 188004), rabbit anti-Glast (Almone lab, AGC-021), mouse anti-Gapdh (Abcam, ab9484), rabbit anti-glutamine synthetase (Abcam, ab49873), mouse anti-glutamine synthetase (Abcam, ab64613), rabbit anti-homer1 (Sysy, 160011), mouse anti-gephyrin (Sysy, 147021). Secondary antibodies conjugated to Alexa488, Alexa568 and Alexa647 were purchased from Molecular Probes (Life Technologies). Nuclear DNA was stained with DAPI (4′,6-diamidino-2-phenylindole, 1 mg/ml, Sigma-Aldrich, 10236276001).

### Transcardiac perfusion fixation of mouse brains and immunohistochemistry analysis

Mice were anesthetized with an injection of 40 to 80 mg/kg of pentobarbital. Then transcardiac perfusion was performed with cold 0.1 M phosphate buffered saline (pH 7.4) (PBS) containing 0.0025 g/50 ml heparin solution following by 4% formaldehyde (FA) in phosphate buffer (Gage et al., 2012). Dissected brains were post-fixed in buffered 4% FA overnight, infiltrated stepwise with 10%, 20% and, 30% sucrose in PBS, and embedded in Tissue-Tek OCT before frozen in melting isopentane [37]. 16 μm thick cryosections and placed onto Poly-L-Lysine (Sigma-Aldrich, P4832-50ML) coated coverslips. After permeabilization in 0.2% Triton X-100 for 10 min (Sigma-Aldrich, 102533092) in PBS, immunostaining performed as previously described [38]. Fluorescence intensity immunoreactivity in mouse hippocampi was analyzed using a build-in ImageJ/Fiji plugin (https://fiji.sc/).

### Morphometric analysis of hippocampal astrocytes

The morphology of astrocytes and neurons was quantified as previously published [39]. In brief, cryosections through the hippocampus of mice were stained for astrocytes by anti-Gfap and counterstained with DAPI. Hippocampal subregions were identified by the Allen mouse brain atlas (https://mouse.brain-map.org/) (Fig. S1). Z-stacked images were maximum intensity projected, converted to 8-bit using ImageJ/Fiji, and pre-processed with an FFT bandpass filter (filter up to 5 pixels, down to 40). Individual astrocytes were cropped, skeletonized, and quantified using AnalyzeSkeleton(2D/3D) and FracLac analysis plugins [40].

### Astrocyte number analysis

Gfap and Sox9 positive astrocyte numbers were counted in different hippocampal subregions in immunostained cryosections of mice brains. Gfap and DAPI positive cells were marked with the image counter plugin of ImageJ/Fiji. For the analysis of Sox9-positive cells, images were converted to 8-bit and “huang dark” threshold was applied, converted to binary, and watershed was applied. Particles analysis was performed with the sizes defined as 100 to infinity and circularity was defined as 0.4 to 1.00. Counted cells were exported and processed in GraphPad prism.

### Mouse hippocampi transcriptome analysis

PN (postnatal day) 40 WT and Adgrv1/del7TM mice were sacrificed by cervical dislocation. The hippocampus was dissected from the brain and flash-frozen using liquid nitrogen. mRNA was isolated according to the instructions of the Qiagen RNeasy Mini kit. The quality and quantity of RNAs were measured with a Nanodrop 2000 spectrophotometer (Thermo Fischer Scientific). Sequencing was performed with Illumina NovaSeq 6000 Sequencing Systems (Novogene). Analysis was performed using Conda package manager v.23.5.2 [41]. All packages were installed in separate environments and an analysis pipeline was written in Snakemake v.7.32.3 [42]. First, we performed a quality control analysis of raw paired end reads using FastQC v.0.12.1 (https://www.bioinformatics.babraham.ac.uk/projects/fastqc/). Then we trimmed our reads using Trimmomatic v.0.39 [43] with HEADCROP:15 parameter. After trimming, we performed contamination check analysis using FastQ Screen v.0.15.3 [44] software and reference genomes: *Escherichia coli* strain K-12 (ASM584v2), *Homo sapiens* (GRCh38.p14), *Metamycoplasma orale* strain NCTC10112 (50465_D02-3), *Mus musculus* (GRCm39), *Staphylococcus aureus* (ASM1342v1). During contamination check, we used Bowtie2 v.2.5.1 as an aligner. Reads that did not match any mammalian genome or aligned to *E. coli*, *M. orale* or *S. aureus* were excluded from subsequent analysis. Then, we aligned the filtered reads to the reference mouse genome (GRCm39) using Hisat2 v.2.2.1 [45] software, sorted and recorded them in the bam format using Samtools v.1.17 [46]. To quantify the number of reads that mapped to each gene we used the featureCounts program that included in Subread v.2.0.6 [47] package. In the following analysis we used only genes with more than 5 mapped reads. After filtering files, we performed a differential expression analysis using DESeq2 v.1.40.2 [48]. In further analysis we used only genes with adjusted p-value less than 0.05. Three biological replicates of WT and Adgrv1/del7TM mice were used. However, during the analysis of the raw data, we observed high ingroup variation in Adgrv1/del7TM. Therefore, to identify the differences between WT and Adgrv1/del7TM group, we excluded the outlier biological replicate to decrease ingroup variation.

### Human fibroblast transcriptome analysis

Dermal primary fibroblast lines were expanded from skin biopsies of human subjects (ethics vote: Landesärztekammer Rhineland-Palatinate to KNW). Fibroblasts from the skin biopsies of a 57-year-old male patient with a nonsense mutation in the *ADGRV1* gene (g.[90006848 C > T], R2959*) [49] were kindly provided by Dr Erwin van Wijk (Radboud University Medical Center, Nijmegen). In this patient, Usher syndrome 2 C was clinically confirmed, along with hearing loss, and vision impairment at the Radboud University Medical Center, Nijmegen; however, there was no evidence of epilepsy reported. Fibroblasts derived from the patient and from a healthy individual were maintained in DMEM/10% FBS (Thermo Fischer Scientific, 31966-021) before the RNA isolation, isolated fibroblast RNAs were subjected to RNAseq analysis with Illumina NovaSeq 6000 Sequencing Systems (Novogene) previously described [50].

### Analysis of tandem affinity purification (TAP) data sets and gene ontology (GO) analysis

Data sets obtained by tandem affinity purification (TAP) from HEK293T cells lysates expressing Strep II/ Strep-Tactin tagged ADGRV1 constructs (Fig. S2a, b) were previously published in Knapp et al., 2022 [3]. We categorized the TAP hits by using Gene Ontology (GO) term analysis following by Cytoscape (http://www.cytoscape.org; accessed on 09 September 2023) plugin ClueGO. Functional protein association networks were prepared by using String database v10 [51]. For transmembrane transporter activity related genes in fibroblasts were screened in the web application of the Amigo2 (https://amigo.geneontology.org/amigo/search/bioentity?q=transmembrane%20transporter%20activity).

### Isolation of primary astrocytes

Astrocytes were isolated from newborn female and male sibling mouse pups on PN0, as previously described [6]. Briefly, PN0 mouse pups were dissected, and their brains enzymatically and mechanically dissociated. The resulting single-cell mixtures were plated on Poly-L-Lysine-coated (PLL) flasks and cultured in DMEM/10% FBS/2% penicillin/streptomycin for 7 to 10 days. Upon reaching confluence, flasks were agitated to remove oligodendrocytes and neurons. To separate primary astrocytes from microglia cells, cultures were trypsinized and plated on sequential bacterial-grade dishes. Microglia cells adhered to the dish surfaces, while astrocytes were recovered from the supernatant. Isolated primary astrocytes were cultured for an additional 7 to 10 days in complete growth medium.

### Isolation of primary neurons

Primary neurons were isolated from the mouse hippocampus on embryonic day E18.5. Hippocampal tissues were dissected and kept in ice-cold HBSS buffer. After washing twice with HBSS to remove dead cells, tissues were trypsinized for 5 min at 37 °C. Single-cell solutions were prepared using fire-polished Pasteur pipettes with varying opening diameters. After trituration, 5 ml HBSS was added to stop the enzymatic reaction. Cell suspensions were allowed to settle for 5 min before aspirating the HBSS/trypsin mixture. Cell pellets were then triturated with 2 ml cold HBSS, and cell numbers were counted. Cells were seeded on PLL coated coverslips and cultured in neuron growth medium containing B27 supplement, L-glutamine, penicillin-streptomycin, and glutamate in Neurobasal medium (ThermoFisher Scientific, 21103049).

### Astrocyte-neuron co-culture

Primary astrocytes were cultured in PLL coated coverslips in a 6 well plate for 2 days at a density of 3 × 10^5^ cells per well in DMEM/10% FBS/2% penicillin/streptomycin medium. After two days of cultivation, primary neurons were isolated and seeded on top of the lawn of astrocytes at a density of 3 × 10^5^ neurons per well. In these co-cultures, the DMEM medium was replaced by neuron growth medium.

### Immunocytochemistry

Primary astrocytes and neurons were processed for immunocytochemistry as previously described [4, 8]. In brief, the cells were gently washed with PBS and fixed with 2% FA in PBS for 10 min. After fixation, cells were washed with PBS and permeabilized by using PBSTX (0.2% Triton X) for 20 min. Permeabilized cells were blocked with 0.1% ovalbumin, 0.5% fish gelatine and incubated with primary antibodies overnight at 4 °C. After PBS washing secondary antibodies and DAPI incubated for 1 h. Coverslips were mounted in Mowiol (Roth) and stored in 4 °C until the imaging.

### Western blot analyses

Protein lysates from tissue and primary cells were prepared as previously described [7] and densitometry analysis was performed by using an Odyssey imaging system (LI-COR Biosciences).

### RFP-TRAP

Immunoprecipitation assays were performed using RFP-Trap^®^ agarose beads (ChromoTek) according to the manufacturer’s instructions. In brief, HEK293T cells were transfected with either the RFP-ADGRV1_CTF (Uni-Prot ID Q8WXG9-1, aa 5891–6306) or RFP-only constructs for 48 h. Transfected cells were lysed using Triton X-100 lysis buffer (50 mM Tris–HCl pH 7.5, 150 mM NaCl, 0.5% Triton X-100) supplemented with a protease inhibitor cocktail (PI mix; Roche). A total of 10% of the HEK293T lysate was reserved as input sample. The remaining HEK293T lysate was then combined with lysate of primary mouse astrocytes derived from mouse brains or hippocampal lysate, thereby facilitating the co-immunoprecipitation of endogenous murine Glast with RFP-tagged human ADGRV1_CTF. Subsequently, the combined lysates were incubated with pre-equilibrated RFP-Trap^®^ beads for 2 h at 4 °C under constant rotation. Following the incubation period, the beads were washed with dilution buffer (10 mM Tris–HCl pH 7.5, 150 mM NaCl, 0.5 mM EDTA). Bound proteins were then eluted using SDS sample buffer. The eluted proteins were then subjected to SDS-PAGE followed by Western blotting for further analysis.

### Glutamate uptake and glutamate dehydrogenase (GDH) activity assays

Glutamate uptake assays were performed as previously described [24]. Briefly, primary astrocytes were seeded at a density of 2 × 10^5^ cells per well of 12 well plate. After 72 h in DMEM medium, astrocytes were treated with serum free Opti-MEM medium for 2 h. Opti-MEM was removed by 3 PBS washes. Cells were exposed to 100 or 200 µM Glutamate solution in Hank´s Balanced Salt Solution (HBSS) containing Ca^2+^ buffer for 4 h. Cell culture supernatants were analyzed in a Glutamate assay kit (Sigma-Aldrich, MAK004) following the manufacturer’s instructions. GDH activities in primary astrocytes were determined with glutamate dehydrogenase activity assay kit following with the manufacturer´s instructions (Abcam, ab102527).

### “Stachel” peptide synthesis

The “Stachel” peptide (SVYAVYARTDN) mimicking the tethered agonist of ADGRV1 [3] and a randomized control peptide (ATSVRYDNAYV) were synthesized from C- to N-terminus by solid phase peptide chemistry, HPLC purified and analyzed by mass spectrometry (Chempeptide limited). Lyophilized peptide was solubilized in 100% DMSO and diluted in 80% ddH_2_0 and 20% HNO_3_ (20% v/v) to obtain a 10 mM stock solution.

### Live cell imaging and “Stachel” peptide application

Glutamate was monitored in primary astrocytes using the fluorescent reporter for glutamate pAAV.GFAP.iGluSnFR3.v857.GPI (Addgene plasmid # 178338) which was a gift from Dr Kaspar Podgorski [52]. Images were acquired by Nikon eclipse Ti2 microscope (Nikon Instruments Inc) equipped with CSU-W1-T2 automatic Spinning Disk Confocal Scanning unit with 63x water objective (Yokogawa Electric Corporation). Dyes were excited with a 488 nm laser. The exposure time was kept at 250 ms and image sequences were obtained every 700 milliseconds for a total of 300 s. 1 mM “Stachel” peptide applied to the HBSS medium to evoke the Adgrv1 signaling at 98th sec of imaging. Fluorescence values were normalized to the first 98 frames of images (F_0_). Calculation of intensity changes was done by following formula (F-F_0_)/ F_0_.

### Morphometric analysis of neurons

To analyze neuronal dendritic complexity, Sholl analysis method was used as previously described [53]. Briefly, images of Map2 positive neurons were exported to ImageJ/Fiji and converted to 8-bit images and “IsoData” thresholding was applied to images. Thresholded images were converted to Mask and fill hole function applied. Binarized images were loaded into the SNT plugin in ImageJ/Fiji. Branches of neurites were selected and semi-autonomous branch detection option of SNT used. Intersection numbers were exported to Excel and Sholl plots were generated using R Studio.

### Synaptic puncta analysis in cultured neurons

Primary neurons isolated from WT and Adgrv1/del7TM mice hippocampi were cultured for 14 days. For the quantification of the number of synaptic puncta along the Map2 positive dendrites, SynapCountJ and NeuronJ Fiji/ImageJ plugins were used [54]. Briefly, dendrites from individual neurons were tracked using NeuronJ plugin and dendritic structures saved as a tab-limited text file (Txt). Next, dendritic branch information and images with synaptic channels were uploaded to SynapCountJ plugin. Images were manually thresholded and threshold values kept constant throughout the analysis. Synaptic densities were calculated per 100 μm dendritic length. Synaptic puncta sizes were analyzed with another Fiji/ImageJ plugin Synapse Counter [55]. Images were thresholded with Triangle and synaptic particle size was limited from 20 px^2^ to 400 px^2^ using the batch mode function of the plugin.

### Microscopy

Images were acquired using a Leica DM6000B Microscope (Leica-Bensheim) or a Nikon eclipse Ti2-E microscope (Nikon Instruments Inc) equipped with CSU-W1-T2 automatic Spinning Disk Confocal Scanning unit (Yokogawa Electric Corporation).

### Statistical analysis

Statistical analyses were performed with Graphpad Prism 9.0 software (GraphPad Software Inc.). Differences between two sets of data were assessed via a two-tailed Student’s t-test. In the case of multiple group comparisons, one way ANOVA was employed, followed by either Dunnett’s multiple comparison tests or Sidak’s multiple comparison tests, chosen based on the specific data being compared. Significance levels were defined as *p < 0.05, **p < 0.01, and ***p < 0.001. The bar plots display means with standard deviations (mean ± SD). The box plots exhibit the median (central line), while the edges of the boxes represent the interquartile range (25th -75th percentile). The whiskers depict the range for the upper 25% and lower 25% of the data points.

## Results

### Adgrv1 controls astrocyte morphology in the mature mouse hippocampus

**Fig. 1:**
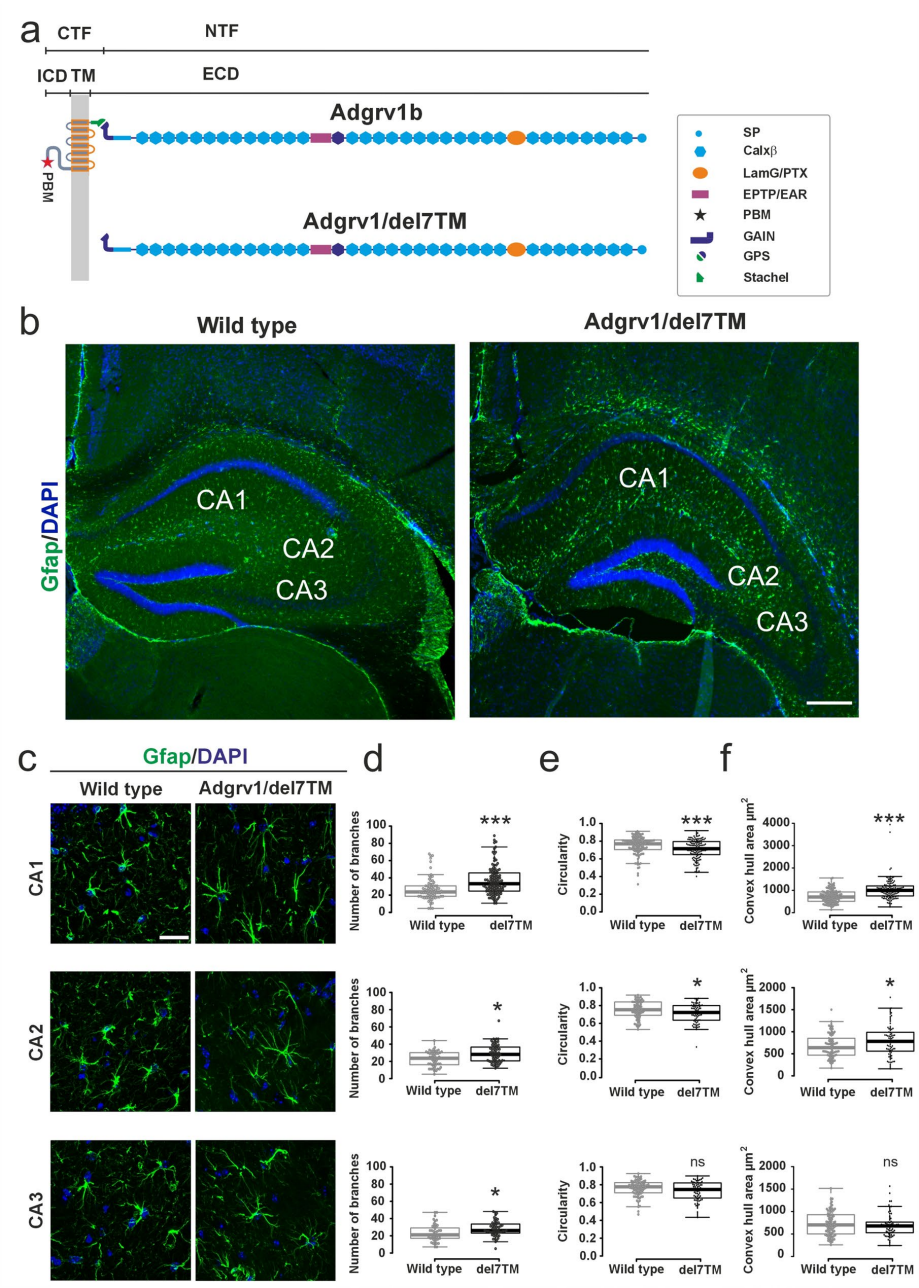
Morphological analysis reveals differences between astrocytes in WT and Adgrv1/del7TM mouse hippocampi. **a** Domain structures of ADGRV1 full length and Adgrv1/del7TM proteins. The cleavage of the full-length molecule from the highly conserved GPCR proteolytic site (GPS) in the GAIN (autoproteolysis-inducing) domain results in relative short C-terminal fragment (CTF) which contains 7-transmembrane (7TM) and intracellular domain (ICD) and extra-large N-terminal fragment (NTF). Extra-large N-terminal fragment contains calcium binding Calxß repeats, seven epilepsy-associated/Epimptin like (EAR/EPTP) repeats, and pentaxin/laminin G-like domain (Lamg/PTX). Adgrv1/del7TM mice carry a nonsense mutation in V2260* which results in STOP codon and leads to deletion 7TM and ICD domains. **b** Overview of hippocampal sections from WT and Adgrv1/del7TM hippocampus through the CA (*Cornu ammonis*) 1, CA2 and CA3 subregions of the hippocampus stained for Gfap and for nuclear DNA by DAPI. **c** Anti-Gfap stained astrocytes in the three different CA1-3 regions of the hippocampus and the quantification of **d** branching, **e** circularity and **f** convex hull areas. 3 consecutive sections were analyzed from *n* = 3 animals per group of mice. In total 150–160 cells from CA1, 80–85 cells from CA2 and 75–85 cells from CA3 were used for the quantification. Scale bar: 25 μm. Statistics: two-tailed Student´s t test; *p ≤ 0.05, **p ≤ 0.01, ***p ≤ 0.001

We have previously shown that the morphology of primary astrocytes derived from brains of Adgrv1/del7TM mice is altered [4]. To examine the astrocyte morphology in situ, we stained cryosections of the hippocampus of mature Adgrv1/del7TM and control wild-type (WT) mice for the astrocyte marker glial fibrillary protein (Gfap) (Figs. 1b, and S1). We determined and quantified the branching, the circularity, and the convex-hull area of astrocytes in the different subregions of the hippocampus (Fig. 1c). The number of branches in astrocytes was significantly increased in all three *Cornu ammonis* regions of the hippocampus (CA1, CA2, and CA3) in Adgrv1/del7TM compared to WT (Fig. 1d, e). The circularity (polarization) and convex-hull area of the astrocytes were significantly reduced in CA1 and CA2, but not in CA3 (Fig. 1e, f). These data suggest that the lack of Adgrv1 results in morphological changes in astrocytes, predominantly in the CA1 region of the hippocampus.

### Loss of astrocytes in hippocampus regions of Adgrv1/del7TM mice

**Fig. 2:**
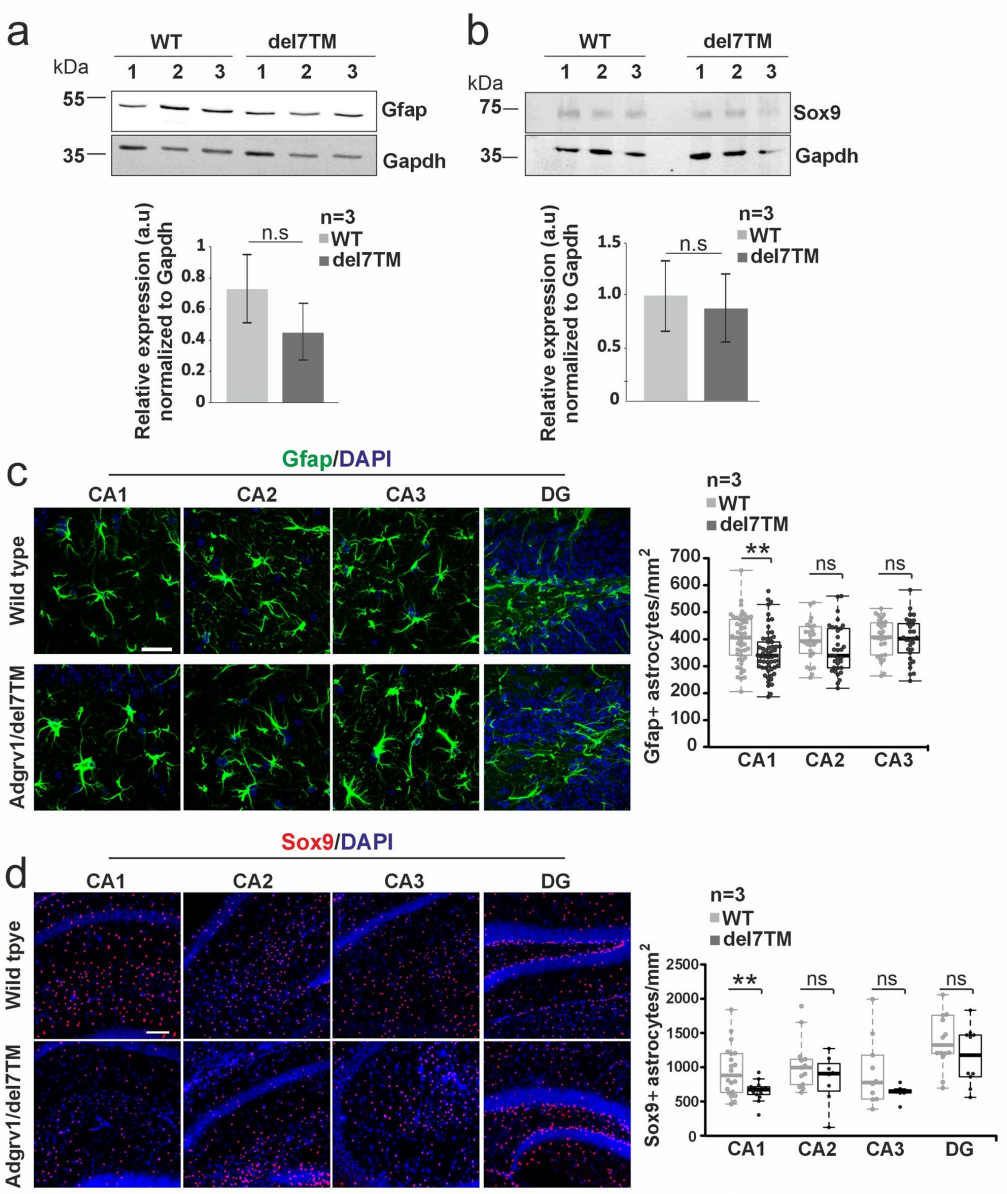
Analysis of the abundance of astrocytes in the hippocampus of Adgrv1/del7TM and wild type mice. **a**, **b** Quantitative Western blot analyses of the astrocyte marker proteins Gfap **a** and Sox9 **b** demonstrate the decrease of the expression of both proteins in the hippocampus of Adgrv1/del7TM mice. **c**,** d** Immunofluorescence staining of Gfap **c** and Sox9 **d**, counterstained by DAPI for nuclear DNA in cryosections through the hippocampus of Adgrv1/del7TM and WT control mice. Quantification of Gfap-positive and Sox9-positive astrocytes revealed a significant decrease in the number of astrocytes in the hippocampus CA1 region of Adgrv1/del7TM mouse compared to WT. 3 consecutive sections were analyzed from *n* = 3 animals per group of mice. Scale bars: C: 25 μm; D: 200 μm. Statistical evaluation by two-tailed Student´s t test; *p ≤ 0.05, **p ≤ 0.01, ***p ≤ 0.001

Next, we examined whether the number of astrocytes in the hippocampus is altered in Adgrv1/del7TM mice. The number estimated by determining the level of expression of the astrocyte-specific markers Gfap and Sox9 in Western blots of hippocampus tissue (Fig. 2a, b). Quantification of Western blot bands showed a slight decrease of Gfap and Sox9 expression in the hippocampus of Adgrv1/del7TM mice. We subsequently stained cryosections through the hippocampus for Gfap, Sox9, and DAPI and counted immunostained astrocytes in the different regions (Fig. 2c, d). There were no significant changes in the number of astrocytes in the CA2, CA3, and dentate gyrus region, but a significant decrease in the number of astrocytes in the CA1 region of Adgrv1/del7TM hippocampi compared to WT controls.

### Affinity proteomics reveals the interaction of ADGRV1 with proteins enriched in astrocytes

We have previously identified numerous putative interacting proteins of ADGRV1 by affinity proteomics capture approaches based on tandem affinity purifications (TAP) combined with mass spectrometry (Fig. S2a) [3, 56]. Re-analyzing our TAP datasets previously obtained in HEK293T and hTERT-RPE1 cells (PXD042629), we identified 289 proteins which are enriched in the proteome of astrocytes [57] as potential interacting proteins to ADGRV1 (Table S1, subtitle 1: Enriched protein annotations; Fig. S2b). GO term analysis of these astrocyte proteins showed that in total 52 proteins are clustered in the GO term “*Transmembrane transporter activity*” (Table S1, subtitle 2: Biological function analysis; Fig. S2c). Interestingly, five of potentially interacting proteins of ADGRV1 were also found under the GO term *“L-glutamate import”* (Table S1, subtitle 3: Transporters and L-glutamate; Fig. S2c, d), suggesting a link to the glutamate homeostasis, a major function of astrocytes in the CNS. These were the two excitatory amino acid transporters SLC1A1/EAAT3 and SLC1A3/EAAT1/GLAST which are core proteins of the glutamate uptake mechanism in astrocytes. In addition, the three mitochondrial transporters SLC25A12/Aralar, SLC25A13, and SLC25A22 were identified as prey, which are enrolled in glutamate metabolism in the TCA (Table S1, subtitle 3: Transporters and L-glutamate import; Fig. S2d) [58].

### Transcriptome analysis reveals differentially expressed genes related to glutamate homeostasis and epilepsy in the hippocampus of Adgrv1/del7TM mice

**Fig. 3:**
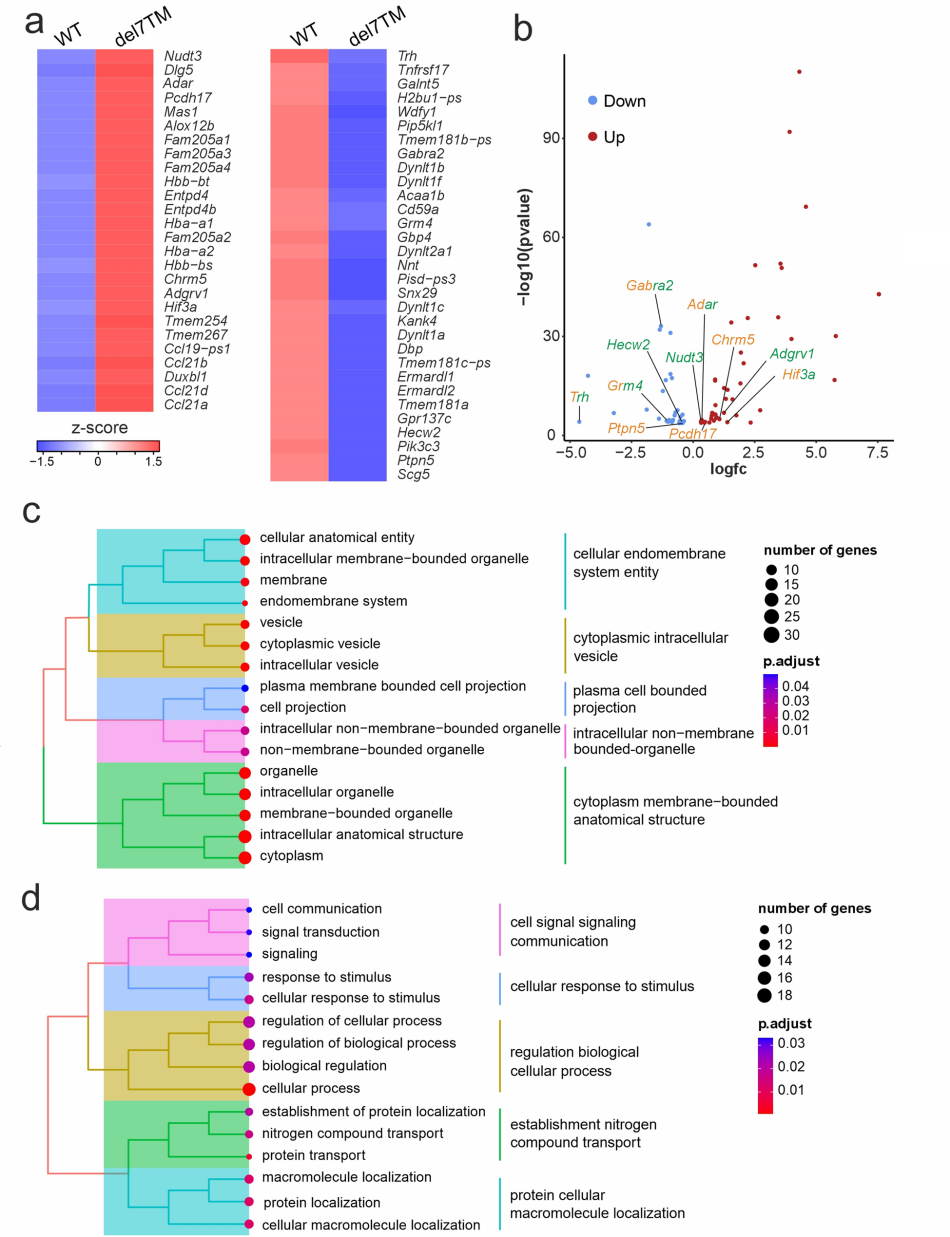
Transcriptome analysis of the hippocampus of Adgrv1/del7TM and WT mice. **a** Heatmap of differentially expressed genes (DEG) in the hippocampus of wild type control (WT) and Adgrv1/del7TM (del7TM) mice. Red, upregulated, and blue, downregulated genes in Adgrv1/del7TM compared to WT. Only adjusted p-values less than 0.05 are shown. **b** Volcano plot of DEGs associated to epilepsy (green) and related to transporter activity (orange) in the hippocampus Adgrv1/del7TM mice. **c** Gene set enrichment (GSE) analysis for the biological function of the DEGs shown in clusters. **d** GSE for the cellular compartment analysis of DEGs. *n* = 3 biological replicates for WT and *n* = 2 biological replicates for del7TM

Next, we performed genome-wide mRNA sequencing from hippocampus tissue dissected from Adgrv1/del7TM and WT mice. The comparison of transcriptomes revealed 80 differentially expressed genes (DEGs) in the hippocampus of Adgrv1/del7TM in relation to WT mice (Table S2). The removal of the non-coding RNAs from the list of DEGs due to incomplete genome annotation [59] resulted in 25 and 31 protein-coding genes being significantly up or down regulated, respectively (Fig. 3a, b; Table S2).

Gene set enrichment analysis (GSEA) of the term “*cellular compartment”* showed that DEGs in the hippocampus of Adgrv1/del7TM mice are associated with “*cellular endomembrane system entity*”, “*cytoplasmic intracellular vesicle*”, “*plasma cell bounded projection*”, “*intracellular non-membrane-bounded organelle”* and “*cytoplasm membrane-bounded anatomical structure*” (Fig. 3C) (date of analysis: 24.08.2023). GSEA of “*biological function”* showed that DEGs in Adgrv1/del7TM hippocampus are associated with functions related to “*cell signaling*”, “*cellular response to stimulus*”, “*regulation of biological processes*”, “*establishment of nitrogen compound transport*” and “*protein cellular macromolecule localization*” (Fig. 3d) (date of analysis: 24.08.2023).

In screens of the Harmonizome 3.0 database [60] (date of analysis: 22.12.2023) we found 6 DEGs associated with epilepsy diseases in Adgrv1/del7TM hippocampus. Of these genes, 4 were found to be upregulated (*Nudt3*, *Adar*, *Adgrv1*, *Hif3a*) while 2 were downregulated (*Trh*, *Grm4*). Interestingly we found that epilepsy associated genes *Adar*,* Hif3a*,* Trh*, and *Grm4* also play a role in glutamate receptor and metabolism regulation. In addition to those DEGs, we identified *Pcdh17*,* Chrm5*,* Gabra2*, and *Ptpn5* which function as glutamate receptor and metabolism regulatory proteins (Fig. 3b).

### Transcriptome analysis of human patient-derived cells confirmed DEGs related to glutamate homeostasis and epilepsy

**Fig. 4:**
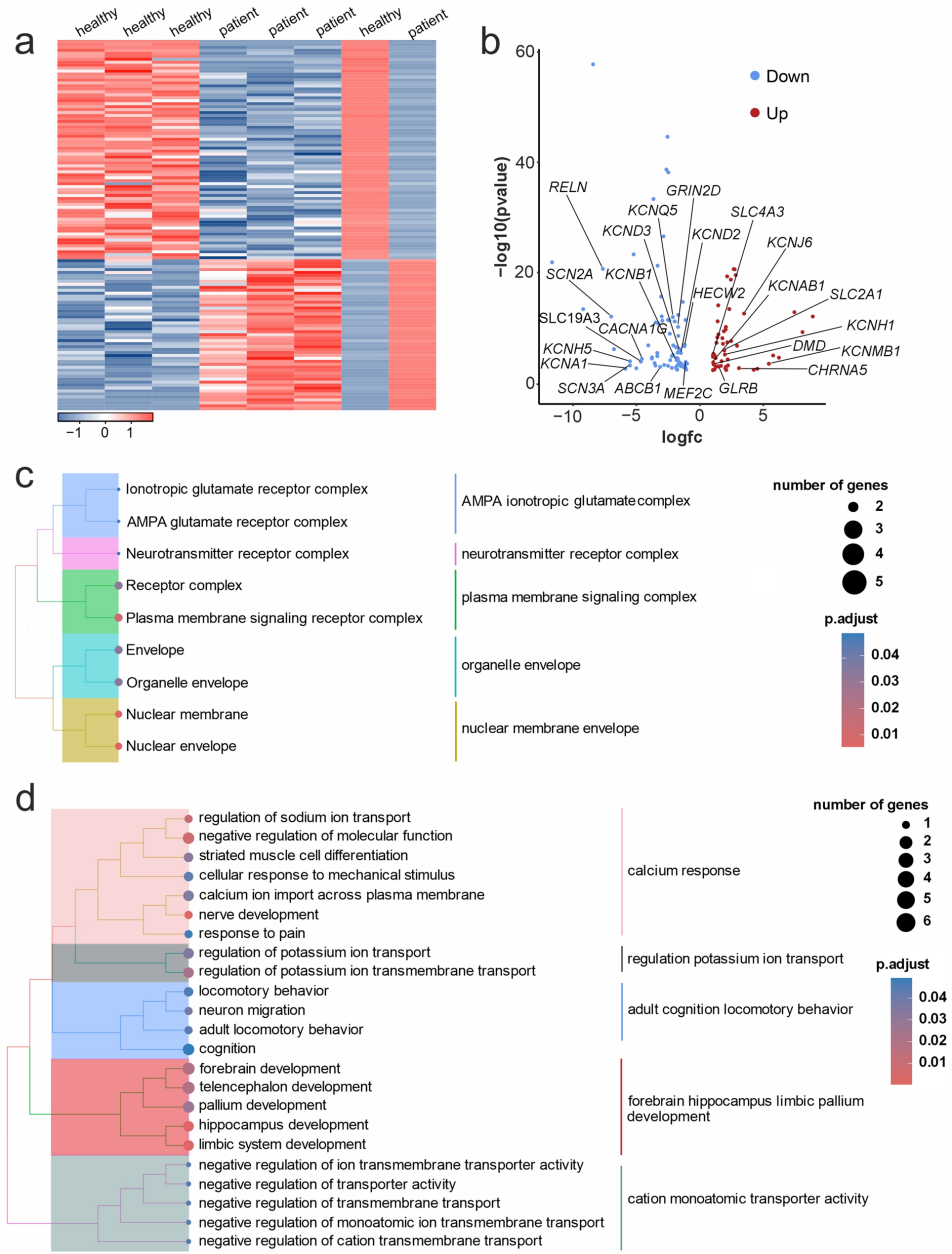
Transcriptome analysis of *ADGRV1*^Arg2959*^ patient-derived fibroblasts compared to fibroblasts from a healthy individuum **a** DEGs of epilepsy-associated and transporter activity genes in patient derived fibroblasts are shown in heatmap analysis. Red color cells indicate upregulated genes and blue color cells indicate downregulated genes. **b** Volcano plot of DEGs shows the genes are common in epilepsy-associated genes and transporter activity related genes in patient-derived fibroblast. **c** Gene set enrichment (GSE) in total 125 DEGs shown in clusters. **d** GSE for “*cellular compartment*” analysis of differentially expressed genes. Only adjusted p-values of less than 0.05 are shown. *n* = 3 replicates for the analysis of healthy and patient fibroblasts

Next, we performed genome-wide sequencing of mRNA derived from dermal fibroblasts of a confirmed USH2C patient and a healthy individual by Illumina platform and pair end reads were mapped and quantified (Fig. 4). Our DEG analysis of the transcriptomes revealed a total of 1,319 DEGs (Table S3, subtitle 1: All DEGs of patient vs. healthy fibroblasts). By GO term analysis applying the web application of the amigo2 and previously reported epilepsy-associated genes [61] we identified in 87 genes related to “transporter activity” and 63 genes related to “epilepsy associated”, respectively (Table S4, subtitle 2: Transporter activity genes, subtitle 3: Epilepsy associated genes) (https://amigo.geneontology.org/amigo) (Fig. 4a, b). Notably, 24 of these DEGs (15 up-regulated and 9 down-regulated) were common to both categories. These genes are involved in transporter activity and are associated with epilepsy, suggesting that ADGRV1 deficiency may specifically disrupt the transport machinery required for cellular homostases (Fig. 4b, Table S3, subtitle 4: Common in Transporter activity and epilepsy associated gene). For further GSEA analysis, we combined “transporter activity” and “epilepsy associated genes” which was a total number of 125 DEGs. Of these DEGs, 74 genes were up- and 51 genes were downregulated (Figs. 4a, S4, Table S3, subtitle 5: DEGs in epilepsy and transport). A comparison with the Adgrv1/del7TM mouse hippocampus revealed that the solute carrier proteins SLC2A1, SLC4A3, and SLC19A3 were present in both transcriptomes. The conservation of these specific transporters across species and cell types suggests that they are core mediators of the metabolic and ionic imbalances driving the Adgrv1 deficiency (Figs. 4a, b, S3).

We additionally performed GSEA on the DEGs which were detected in the patient fibroblasts. The *cellular compartment* GSEA showed that DEGs are related to “*AMPA ionotropic glutamate complex*”, “*neurotransmitter receptor complex*”, “*plasma membrane signaling complex*”, “*organelle envelope*” and “*nuclear membrane envelope*” (Fig. 4c). This indicates that even in fibroblasts, the loss of ADGRV1 mirrors the synaptic signaling defects seen in the brain. GSEA of *biological function* showed that DEGs in patient fibroblast are associated with “*calcium response*”, “*regulation potassium ion transport*”, “*adult cognition locomotory behavior* “*forebrain*,* hippocampus*,* limbic and pallium development*” and “*cation monoatomic transporter activity*” (Fig. 4d). GSEA of the “*biological function”* subclusters showed that DEGs in patient fibroblast are also related to “*nerve development and neuron migration”* as well as the “*development of the brain regions”*, such as the forebrain, hippocampus, and telencephalon (Fig. 4d). Specifically, the dysregulation of potassium and calcium transport genes provides a tangible link to the hyperexcitability and seizure susceptibility observed in patients.

Altogether, these data demonstrate that Adgrv1 deficiency causes a coordinated dysregulation of genes related to glutamate homeostasis and ion transport. By relating these transcriptomic changes to the specific failure of membrane transporters, we propose a mechanism where impaired ionic balance in patient-derived cells reflects the systemic molecular basis for the homeostasis imbalance seen in the central nervous system.

### Glutamate uptake of primary brain astrocytes depends on Adgrv1 activation

**Fig. 5:**
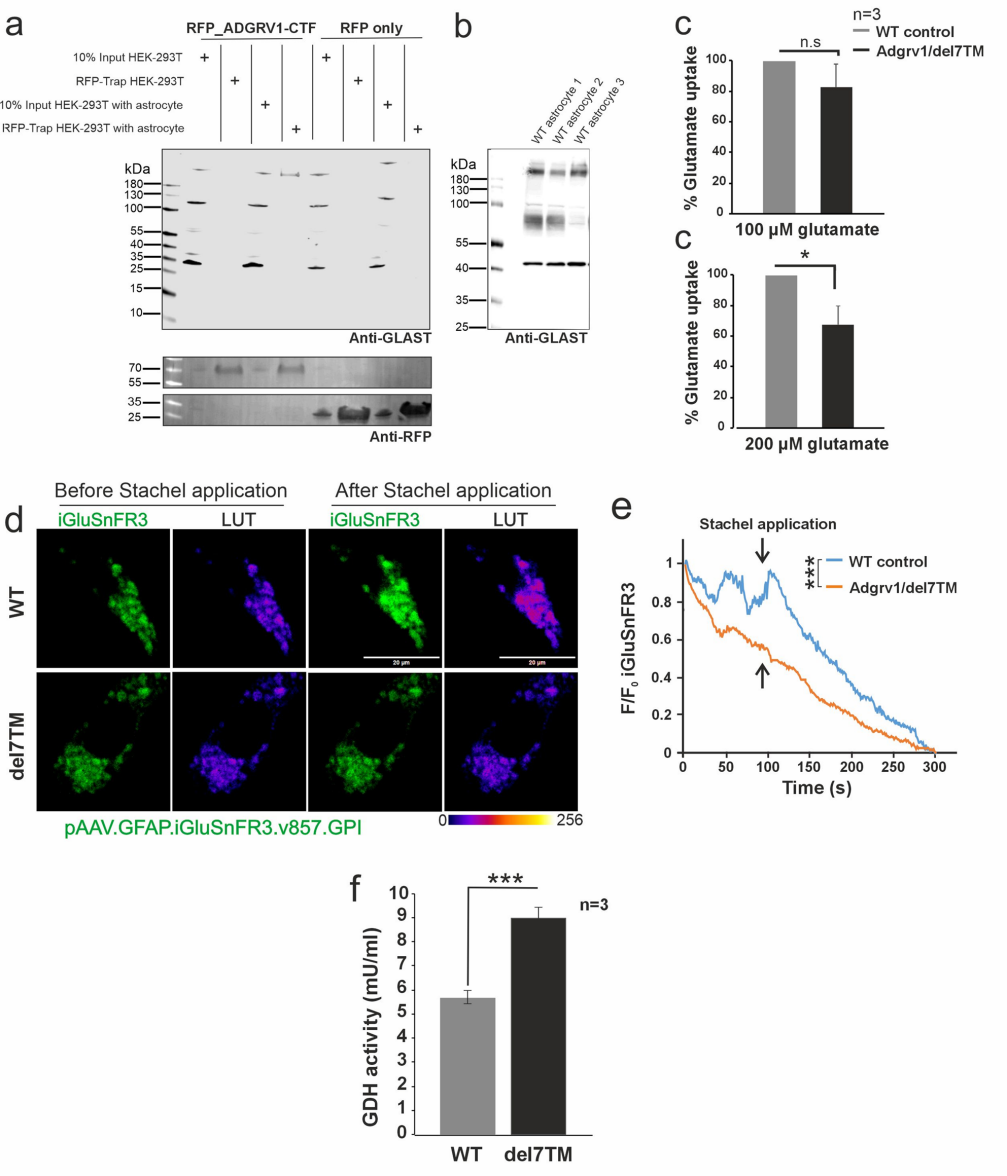
Glutamate uptake is altered in Adgrv1 deficient primary astrocytes. **a** RFP-TRAP pull-down of astrocytic Glast by RFP-ADGRV1_CTF. RFP-Trap pull-down assays were performed using lysates from HEK-293T cells transfected with RFP-ADGRV1-CTF or RFP-only (control) and primary astrocytes. Immunoblotting with anti-GLAST antibodies shows that Glast co-precipitates with RFP-ADGRV1-CTF, but not with the RFP-only control. **b** Astrocytic glast expression evaluated by Western blot analysis indicates monomeric and multimeric presence of Glast **c** Glutamate uptake assay showing a dose-dependent reduction in Adgrv1/del7TM primary astrocytes. **d** Live-cell imaging of pAAV.GFAP.iGluSnFR3.v857.GPI transfected primary astrocytes derived from WT and Adgrv1/del7TM mice. Time-lapse image sequences of the fluorescent glutamate reporter were recorded with a sequence of 700 ms in a time course of 300 s with and without activation by the Adgrv1 Satchel peptide. Left panels (green): Representative fluorescence images of WT and del7TM primary astrocytes expressing the membrane-tethered glutamate sensor pAAV.GFAP.iGluSnFR3.v857.GPI. Images show sensor intensity before and after Adgrv1 activation. Right panels show the same images processed with a 16-color Look-Up Table (LUT) to highlight differences in local glutamate concentrations (Scale: 0-256). Note the sustained high-intensity signal in del7TM astrocytes compared to WT after activation, indicating impaired glutamate clearance. **e** Fluorescence intensity changes calculated with F/F_0_ formula to obtain before and after application. Arrows indicate time point of application of 1 mM “Stachel” peptide for Adgrv1 activation. F/F_0_ iGluSnFR3 intensity changes revealed an increase in WT astrocytes after Stachel peptide application, whereas there was no change observed in Adgrv1/del7TM astrocytes. **f** Glutamate dehydrogenase activity significantly increases in Adgrv1/del7TM astrocytes. For glutamate dehydrogenase activity assay 3 technical and 3 biological replicates were used for the quantification. For iGluSnFR3 intensity analysis *n* = 7 (WT) and *n* = 9 (Adgrv1/del7TM) in *n* = 3 independent experiments were used. Data are represented as mean ± SD. Statistical evaluations were performed for bar plots using two-tailed Student’s t test and using Kruskal-Wallis test for iGluSnFr3 curves*p % 0.05, **p % 0.01, ***p % 0.001. Scale bars: 20 μm

Our omics data analysis suggested that ADGRV1 may play an important role in glutamate homeostasis in hippocampal astrocytes. Among the five candidate interacting proteins, SLC1A3 (Glast) exhibits the highest expression in astrocytes [52]. Anti-Glast Western blots of lysates derived from three independent astrocyte cultures demonstrated the presence of bands related to monomeric Glast and multimeric Glast (Fig. 5b). This confirms the expression of monomeric Glast and the formation of Glast multimers that resist solubilization in murine astrocytes, as previously reported in brain lysates [54, 55]. Next, we validated the potential interaction between ADGRV1 and endogenous Glast. To this end, we expressed RFP-tagged ADGRV1_CTF and RFP alone in HEK293T cells. We combined the protein lysates of these HEK293T cells with lysates derived from wild-type primary astrocytes or hippocampal lysates. Subsequent RFP-TRAP pull downs revealed that Glast multimers from primary murine astrocytes were exclusively co-immunoprecipitated with RFP-ADGRV1_CTF, but not with RFP alone (Fig. 5a). Interestingly, Glast monomers were not co-immunoprecipitated with RFP-ADGRV1_CTF in RFP-TRAPs. This confirms a specific interaction between ADGRV1 and SLC1A3 (Glast) in astrocytes indicated by affinity proteomics (Figs. 5a, Fig. S3).

To confirm the data at a functional level, we accessed glutamate uptake in primary astrocytes in vitro using a glutamate uptake assay introduced by Mahmoud [24]. Primary astrocytes were isolated from Adgrv1/del7TM and WT PN0 mice and incubated with either 100 or 200 µM glutamate in a Ca^2+^ containing cell culture medium. Colorimetric analysis of the supernatants demonstrated the dose-dependent reduction in glutamate uptake by Adgrv1/del7TM astrocytes compared to WT controls (Fig. 5c).

To follow the uptake of glutamate from the media into the primary astrocytes, we used the genetically encoded glutamate reporter GFAP.iGluSnFR3.v857.GPI [52]. This glutamate reporter contains a glycosylphosphatidylinositol anchor that allows binding to glycosylphosphatidylinositols in the extracellular face of the plasma membrane and thereby to monitor changes in glutamate concentration there [62]. However, the reporter was also found to be localized in internal membranes and thus also enables monitoring changes in glutamate concentration in the cell [63]. In the first set of experiments, we confirmed that the glutamate reporter activity can be induced in both primary WT and Adgrv1/del7TM astrocytes by the addition of 10 mM glutamate to the culture medium (Fig. S5a). Next, we activated Adgrv1 using the “Stachel” of ADGRV1, an 11-amino acid peptide SVYAVYARTDN, which we had previously identified as the tethered agonist ADGRV1 [3] to the culture medium of GFAP.iGluSnFR3.v857.GPI expressing primary astrocytes. Activation of Adgrv1 by the “Stachel” peptide led to an increase in reporter fluorescence in primary WT astrocytes, but not in astrocytes derived from Adgrv1-deficient Adgrv1/del7TM (Fig. 5d, e). A control peptide, which consisted of a randomized amino acid sequence and should not activate Adgrv1, did not cause an increase in fluorescence in WT astrocytes (Fig. S5b). Collectively, our data demonstrates that glutamate uptake into primary brain astrocytes depends on the activation of Adgrv1.

### The catabolism of internalized glutamate is increased in Adgrv1/del7TM hippocampal astrocytes

Alternative to its detoxification in the glutamate-glutamine cycle, glutamate can be also metabolized in astrocytes by TCA in mitochondria [64]. Astrocytic mitochondria and redox control are now understood to be dynamic forces in the brain, driving glial-neuronal interactions that regulate core functions like metabolic support, inflammation, and neural circuit longevity [65]. In the TCA, glutamate dehydrogenase (GDH) is the key enzyme, and its activity is a benchmark for glutamate metabolization. Measuring the GDH in cultured astrocytes with a colorimetric assay revealed an almost 1.5-fold increase in GDH activity in Adgrv1/del7TM astrocytes compared to WT astrocytes (Fig. 5d).

### Expression of glutamine synthetase is reduced in hippocampus of Adgrv1/del7TM mice

**Fig. 6:**
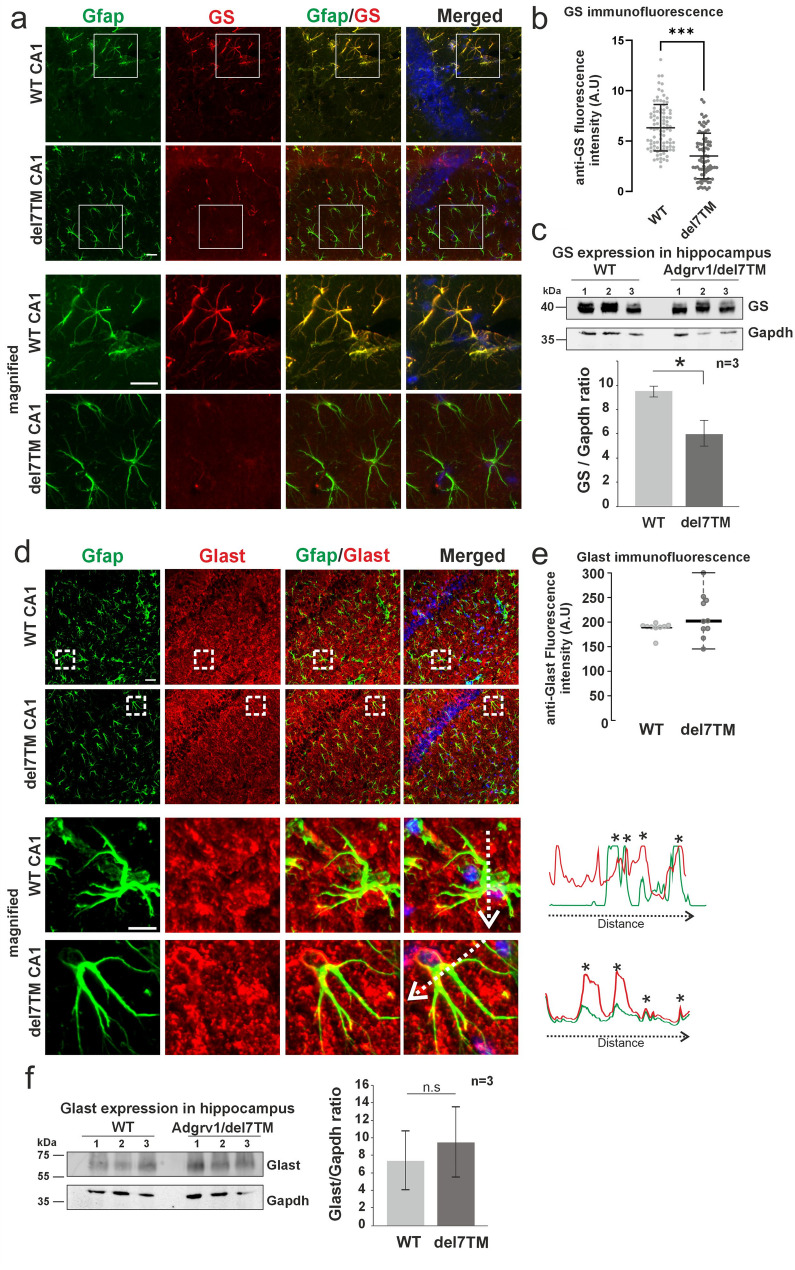
Expression analysis of glutamine synthetase (GS) and Glast in astrocytes of the hippocampus of Adgrv1/del7TM and wild type control mice. **a** Double immunofluorescence staining for GS (red) and for the astrocyte marker Gfap (green) in sections through the CA1 region of the hippocampus counterstained for nuclear DNA by DAPI (blue). Merged images revealed localization and abundant expression of GS in Gfap-positive astrocytes in WT mice which is almost absent in Adgrv1/del7TM mice. **b** Quantification of anti-GS immunofluorescence intensity in in Gfap-positive astrocytes of hippocampal CA1 region reveals a high significant decrease in Adgrv1/del7TM mice. **c** Anti-GS Western blot analysis of hippocampal lysates demonstrates the significant decrease of GS protein expression in Adgrv1/del7TM mice. **d** Indirect immunofluorescence staining for Gfap (green) and glutamate transporter Glast (red) counterstained by DAPI (blue) in the CA1 region of the hippocampus of Adgr1/del7TM (del7TM) and wild type (WT) mice. **e** Quantification of anti-Glast immunofluorescence intensity in Gfap-positive astocytes of hippocampal CA1 region shows slight but not significant increase in Adgrv1/del7TM. *n*=3, number of sections quantified: 3 continuous sections per group. **f** Anti-Glast Western blot analysis of hippocampal lysates of Adgrv1/del7TM and WT mice. Densitometry analysis of anti-Glast bands related to expression of the housekeeping protein Gapdh (A.U) showed a tentative but no significant upregulation of Glast expression in Adgrv1/del7TM. *n*=3 animals and 3 continuous sections per group were used. Statistics: two-tailed Student´s t test, One way-ANOVA test; *p≤0.05, **p≤0.01, ***p≤0.001. Scale bars: 20 µm. Magnified images Scale bars: 200 µm and 20 µm

Next, we analyzed the protein expression of key components of the glutamate-glutamine cycle in astrocytes of the hippocampal CA1 region: the glutamate transporter Glast which imports glutamate from the extracellular space [66] and the glutamine synthetase (GS) catalyzes the glutamine synthesis from glutamate in the cell [67] (Fig. 6). We immunostained cryosections through the hippocampal CA1 region of Adgrv1/del7TM and WT mice for GS, Gfap, and DAPI (Fig. 6a). Confocal microscopy demonstrated bright immunofluorescence of GS in Gfap-positive astrocytes of WT mice, and a greatly reduced fluorescence in astrocytes of Adgrv1/del7TM mice (Fig. 6b). Quantification of anti-GS Western blots of hippocampus reveals a significant decrease in Adgrv1/del7TM mice (Fig. 6c). In contrast, we observed only a minor and not significant increase in Glast expression in the hippocampus of Adgrv1/del7TM neither by immunohistochemistry nor Western blotting (Fig. 6d-f).

### Adgrv1 affects neurite morphogenesis in neurons

**Fig. 7:**
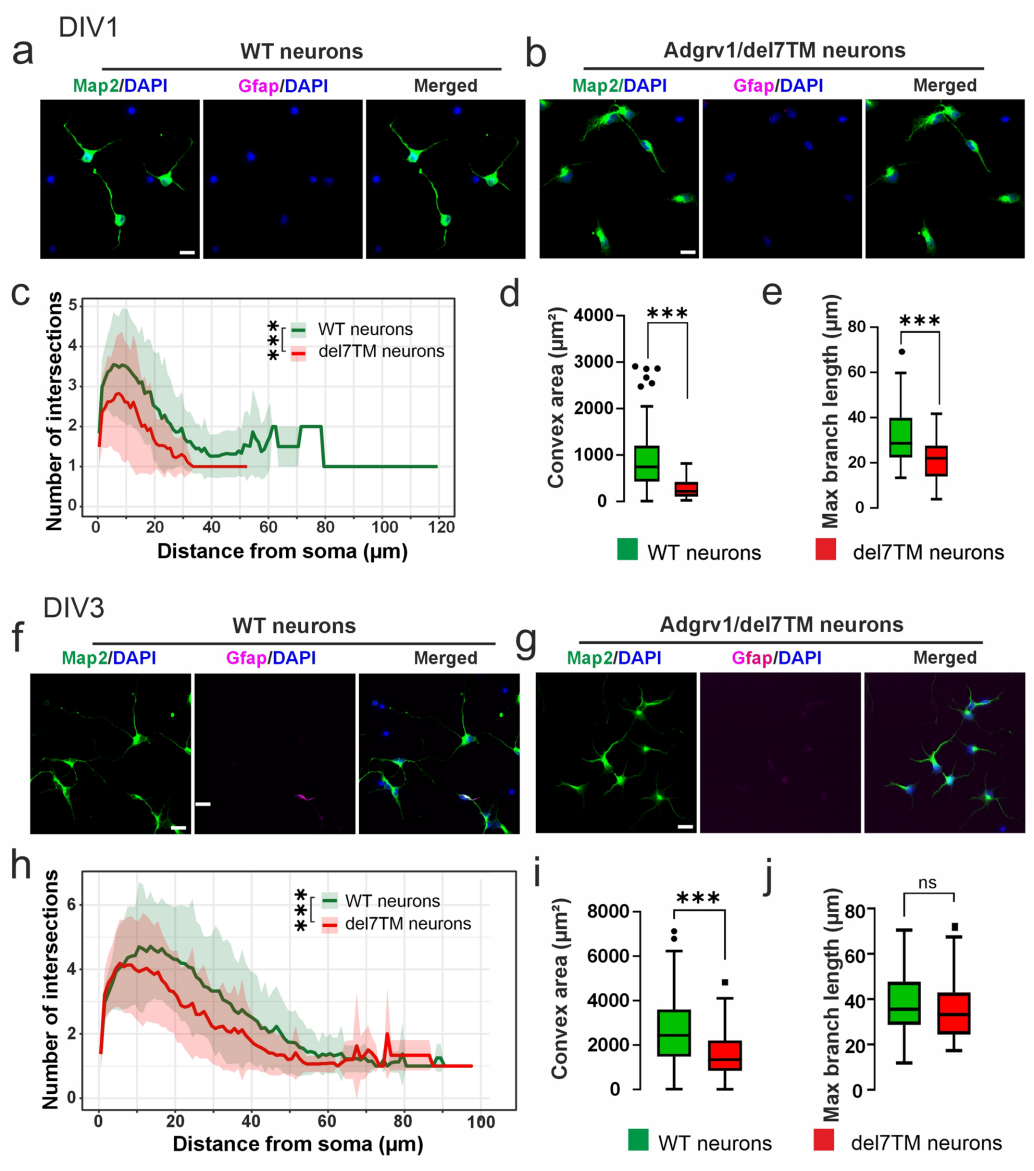
Morphometric characterization of primary neurons derived from WT and Adgrv1 mouse hippocampi. **a**,** b** Primary neurons isolated from the E18.5 WT and Adgrv1/del7TM mouse hippocampi were cultured for 1 day and stained with anti-Map2 (green) and anti-Gfap(magenta) to visualize neurites of neurons and astrocytes, respectively. **c** Sholl analysis method applied for the quantification of intersection numbers of WT (green line) and Adgrv1/del7TM (red line) neuron cultures. A significant decrease in the number of neuronal intersections were observed in Adgrv1/del7TM neurons cultures compared to the WT neuron cultures in day in vitro 1 (DIV1). **d** Analysis of Convex area showed a significant reduction in area of Adgrv1/del7TM neurons compared to WT neurons. **e** Maximum branch length of intersection in Adgrv1/del7TM neurons were also reduced. **f**, **g** WT and Adgrv1/del7TM neurons were cultured for 3 days in vitro (DIV 3). Neurons were identified with anti-Map2 (green) and possible astrocyte contamination were detected with anti-Gfap(magenta) antibodies. **h** Quantification of **i**ntersection numbers by sholl analysis showed that Adgrv1/del7TM neurons have significantly less intersection numbers compared to WT neurons in DIV3. **i** Convex area quantification revealed that reduction in the area of Adgrv1/del7TM neurons persisted in DIV3 compared to WT neurons. **j** Maximum branch length of intersection analysis in DIV3 showed tentative decrease but no significant differences in between Adgrv1/del7TM and WT neurons. 60–70 cells in total for *n* = 3 experiments. Statistics: One way-ANOVA test; *p ≤ 0.05, **p ≤ 0.01, ***p ≤ 0.001. Scale bar: 10 μm

To investigate the effects of Adgrv1 in neurons, we analyzed neuron cultures from brains of Adgrv1/del7TM and WT mice on day 1 in vitro (DIV1) and day 3 in vitro (DIV3) by staining for the neuronal marker Map2 and with DAPI (Fig. 7).

Morphological analysis of neurites in fluorescent microscopy images for neurite intersections (Sholl analysis), convex area, and maximal branch length revealed that all parameters were reduced in Adgrv1/del7TM neurons at both DIV1 and DIV3 (Fig. 7c, h). All quantification analyses revealed significant differences (Fig. 7d, i), except for maximum branch length measurements in DIV3, which shows only a tendency in reduced length in Adgrv1/del7TM neurons (*p* value: 0.70) (Fig. 7j). These findings suggest that Adgrv1 is not only important in astrocyte morphology (Fig. 1c-f) but also has an additional significant influence on neuronal morphogenesis.

### Astrocytic Adgrv1 affects neurite morphogenesis

**Fig. 8:**
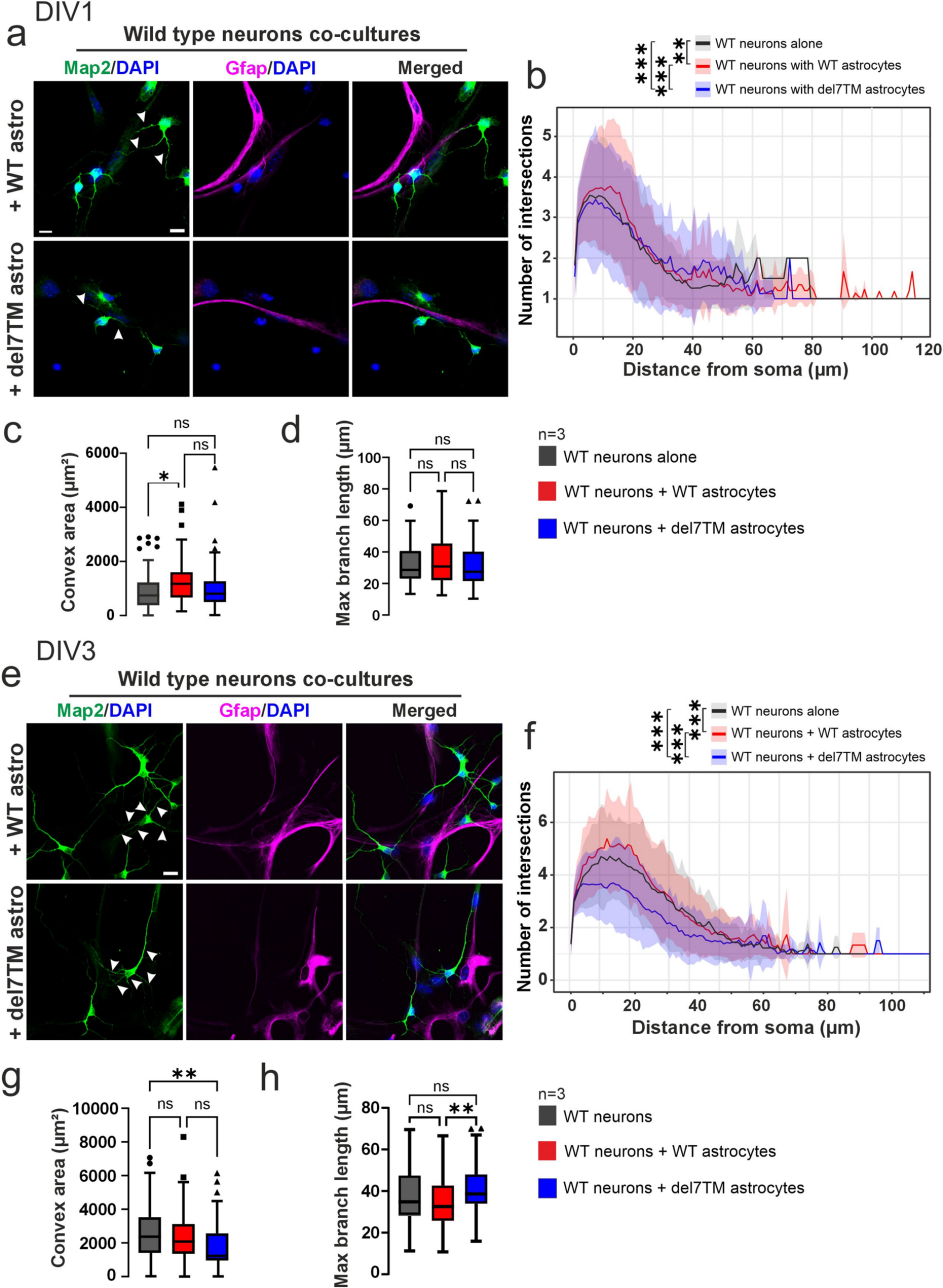
Morphometric characterization of primary neurons derived from WT mouse hippocampi co-cultured with WT and Adgrv1/del7TM astrocytes. **a** Primary neurons isolated from the WT E18.5 mouse hippocampus were cultured alone and together with WT astrocytes (+ WT astro, upper panel) or Adgrv1/del7TM astrocytes (+ del7TM astro, lower panel) for DIV1. Anti-Map2 (green) and anti-Gfap (magenta) were used for the visualization of neuronal neurites and astrocytes, respectively. Arrow heads indicate the neurites of primary neurons. **b** Sholl analysis revealed that co-culturing of WT neurons with WT astrocytes (red line) resulted in significant increase of intersection numbers of WT neurons. On contrary, co-culturing of WT neurons with Adgrv1/del7TM astrocytes (blue line) showed slight but significant decrease compared to WT neuron cultures (black line). **c** Convex area analysis showed a notable increase in area of WT neurons co-cultured with WT astrocytes. However, no significant changes were observed in co-cultures with Adgrv1/del7TM astrocytes. **d** Maximum branch length of intersection in WT neurons did not change on both co-cultured together with WT and Adgrv1/del7TM astrocytes. **e** Primary neurons isolated from the WT E18.5 mouse hippocampus were cultured alone and together with WT astrocytes (+ WT astro, upper panel) or Adgrv1/del7TM astrocytes + del7TM astro, lower panel) for DIV3. Anti-Map2 (green) and anti-Gfap (magenta) were used for the visualization of neuronal neurites and astrocytes, respectively. Arrow heads indicate the neurites of primary neurons. **f** Total number of the intersections in WT neurons co-cultured with WT astrocytes (red line) showed the highest neurite numbers in DIV3 similar to DIV1 cultures. The analysis revealed that compared to WT neurons cultures (black line) and WT neurons with WT astrocyte cultures (red line), co-culturing of WT neurons with Adgrv1/del7TM astrocytes (blue line) resulted in the lowest intersection numbers in DIV3 of culture. **g** Convex area quantification showed a significant decrease in WT neuron areas co-cultured with Adgrv1/del7TM astrocytes in DIV3. However, no significant changes were observed in the WT neuron area when co-cultered with WT astrocytes. **h** Maximum branch length of intersections in WT neurons did not change in both co-cultures together with WT and Adgrv1/del7TM astrocytes in DIV3. However, analysis revealed that WT neurons with WT astrocytes co-cultures have significantly smaller areas compared to WT neurons with Adgrv1/del7TM astrocytes. 60–70 cells in total for *n* = 3 experiments. Statistics: One way-ANOVA test; *p ≤ 0.05, **p ≤ 0.01, ***p ≤ 0.001. Scale bar: 10 μm

**Fig. 9:**
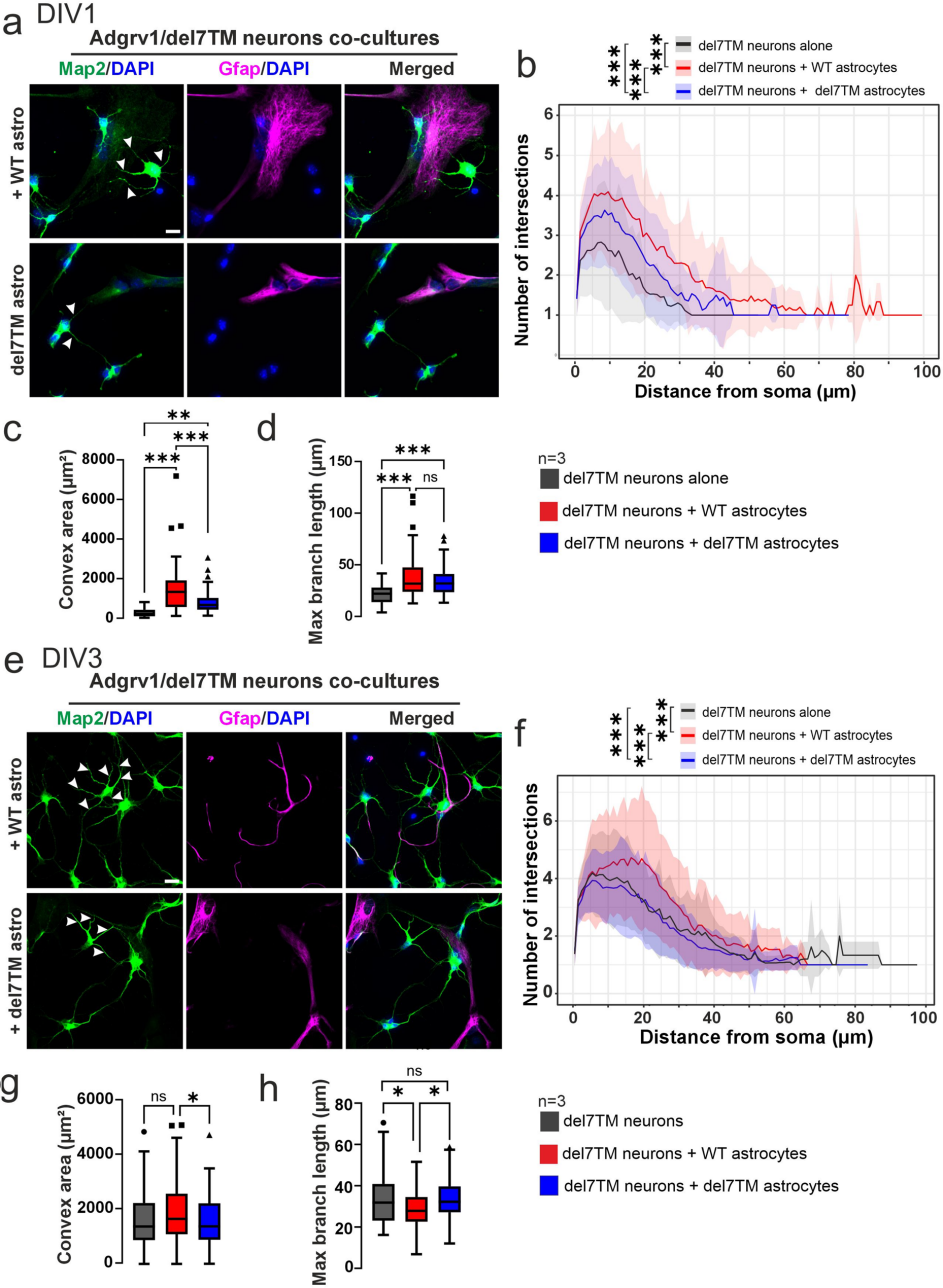
Morphometric characterization of primary neurons derived from Adgrv1/del7TM mouse hippocampi co-cultured with WT and Adgrv1/del7TM astrocytes. **a** Primary neurons isolated from the Adgrv1/del7TM E18.5 mouse hippocampus were cultured alone or together with WT (+ WT astro, upper panel) or Adgrv1/del7TM astrocytes (+ del7TM astro, lower panel). Anti-Map2 (green) and anti-Gfap (magenta) were used for the visualization of neuronal neurites and astrocytes, respectively. Arrow heads indicate the neurites of neurons. **b** Sholl analysis of Adgrv1/del7TM neurons cultures (black line) revealed the least intersection numbers. Co-culturing of the Adgrv1/del7TM neurons with WT (red line) or Adgrv1/del7TM (blue line) resulted in significant increase in intersection numbers. The most prominent increase in intersection numbers was observed in Adgrv1/del7TM neurons co-cultured with WT astrocytes. **c** Co-cultures of Adgrv1/del7TM neurons with WT or Adgrv1/del7TM astrocytes significantly increased the area of Adgrv1/del7TM neurons. The most prominent increases in the Adgrv1/del7TM neuron areas were observed in co-cultures with WT astrocytes. **d** Maximum branch length of intersection in Adgrv1/del7TM neurons significantly increased in both co-cultured together with WT and Adgrv1/del7TM astrocytes in DIV1. **e** Primary neurons isolated from the Adgrv1/del7TM E18.5 mouse hippocampus were cultured alone and together with WT astrocytes (+ WT astro, upper panel) or Adgrv1/del7TM astrocytes + del7TM astro, lower panel) for DIV3. Anti-Map2 (green) and anti-Gfap (magenta) were used for the visualization of neuronal neurites and astrocytes, respectively. Arrow heads indicate the neurites of primary neurons. **f** The highest intersection numbers were observed in Adgrv1/del7TM neurons when co-cultured with WT astrocytes (red line). Adgrv1/del7TM neurons co-cultured with Adgrv1/del7TM astrocytes (blue line) showed significantly lowest intersection numbers compared to both Adgrv1/del7TM neuron cultures (black line) and Adgrv1/del7TM neurons/WT astrocyte co-cultures in DIV3. **g** Co-culturing the Adgrv1/delTM neurons with both WT and Adgrv1/del7TM astrocytes did not change the area of Adgrv1/del7TM neurons. However, significant reduction was observed in Adgrv1/del7TM neuron and Adgrv1/del7TM astrocyte co-cultures compared to co-cultures with WT astrocytes in DIV3. **h** Maximum branch length of intersections in Adgrv1/del7TM neurons co-cultured with WT astrocytes showed significant reduction compared to Adgrv1/del7TM neuron and Adgrv1/del7TM neuron co-cultured with Adgrv1/del7TM astrocytes in DIV3. 60–70 cells in total for *n* = 3 experiments. Statistics: One way-ANOVA test; *p ≤ 0.05, **p ≤ 0.01, ***p ≤ 0.001. Scale bar: 10 μm

Astrocytes cooperate with neurons and support them in a variety of ways to maintain and nurture the neuronal microenvironment and help to control neuronal migration during development [68]. To investigate the effects of Adgrv1 in astrocytes on neurons, we analyzed cocultures of primary astrocyte and neurons from brains of Adgrv1/del7TM and WT mice at DIV1 and DIV3 and identified neurons and astrocytes with immunostaining of Map2 and Gfap, respectively (Figs. 8 and 9).

Sholl analysis of the fluorescence images indicated a significant increase in the number of neurite intersections in WT neurons when co-cultured with WT astrocytes at both DIV1 and DIV3, indicating a synergistic effect on neuronal development (Fig. 8b, f). In contrast, co-culturing WT neurons with Adgrv1/del7TM astrocytes led to fewer neurite intersections at both stages, with the most pronounced effect at DIV3 (Fig. 8b, f). Furthermore, the convex area of neurites in WT neurons was significantly increased by WT astrocytes at DIV1 but not at DIV3, suggesting a temporal influence on neurite morphology (Fig. 8c, g). Interestingly, DIV3 cultures with Adgrv1/del7TM astrocytes exhibited a decrease in convex area compared to WT neuron cultures alone, highlighting the impact of Adgrv1 deficiency on neurite development (Fig. 8c, g). However, the maximum branch length analysis revealed no significant differences in WT neurons when co-cultured with either WT or Adgrv1/del7TM astrocytes at DIV1 and DIV3 (Fig. 8d, h).

Next, we explored the role of astrocyte Adgrv1 expression on Adgrv1-deficient neurons. Sholl analysis revealed a substantial increase in neurite numbers in the presence of WT astrocytes at both DIV1 and DIV3 (Fig. 9b, f). Additionally, WT astrocytes significantly increased the convex area of neurites in WT neurons at DIV1, though not at DIV3, indicating a dynamic interplay between astrocytic Adgrv1 expression and neurite morphology (Fig. 9c, g). Moreover, maximum branch length analysis of Adgrv1/del7TM neurons showed an initial increase at DIV1 followed by a significant decrease at DIV3 in the presence of WT astrocytes (Fig. 9d, h). However, Adgrv1/del7TM astrocytes showed an opposite effect on Adgrv1/del7TM neurons, namely an increase of the number of intersection numbers and of convex area of neurites at DIV1 (Fig. 9b, c) but inducing a significant decrease at DIV3 cultures (Fig. 9f, g).

In summary, Adgrv1 in astrocytes supports the beneficial role of astrocytes in neurite morphogenesis during neuronal development and the dysfunctions of Adgrv1-deficient astrocytes exacerbate defective neurite morphogenesis in Adgrv1-deficient neurons.

### Adgrv1 functions in the astrocyte-neuron crosstalk affecting synaptogenesis

**Fig. 10:**
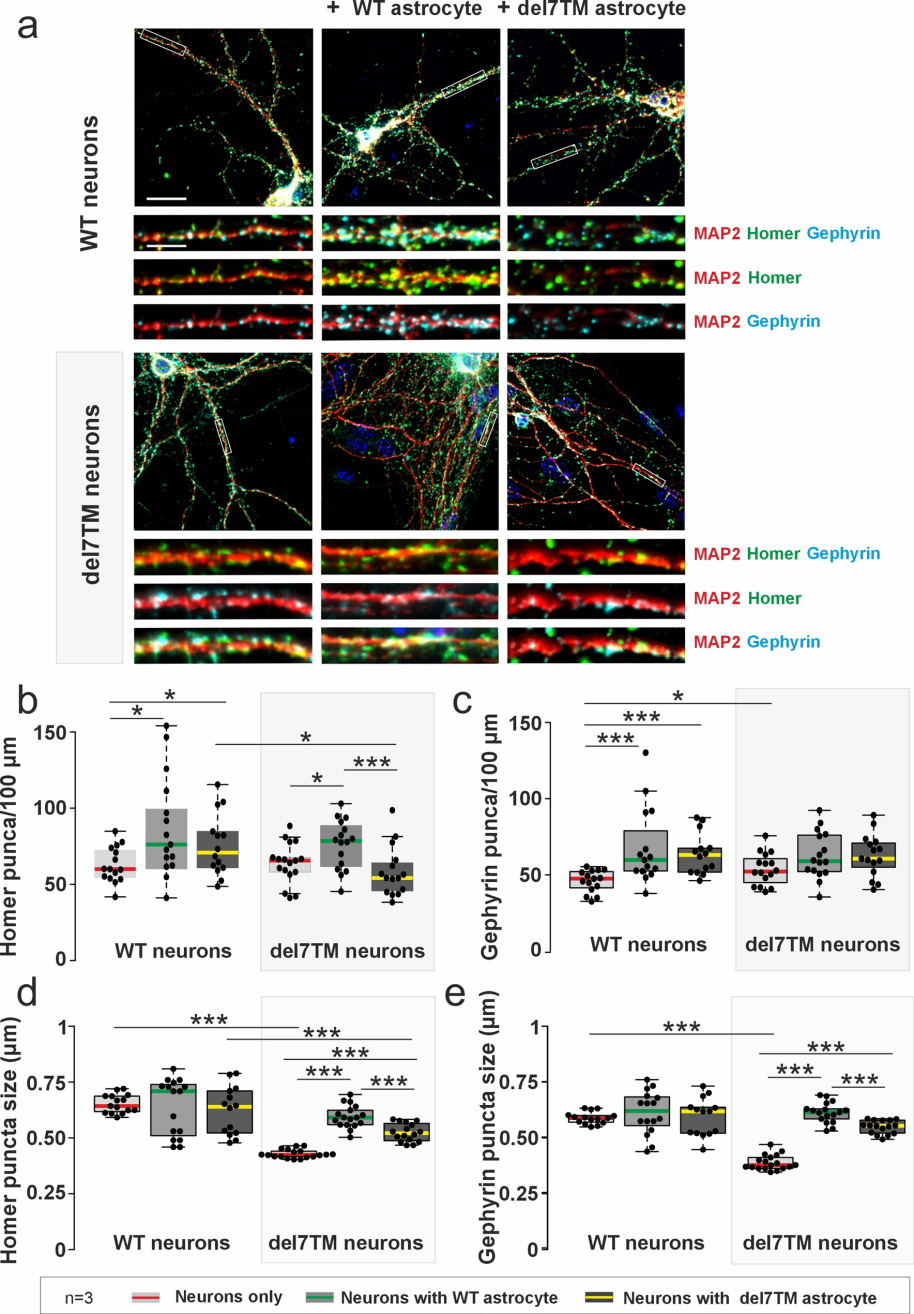
Co-culturing of WT and Adgrv1/del7TM neurons with WT astrocytes increases synaptic numbers and sizes. **a** Hippocampal neurons from WT and Adgrv1/del7TM mice were immunolabelled with anti-Homer (Green), anti-Gephyrin (Cyan) and anti-Map2 (red). Nucleus was counter-stained with DAPI. **b** Quantification of excitatory post-synaptic puncta revealed similar Homer puncta in WT and Adgrv1/del7TM neuron cultures. WT astrocyte and neuron co-cultures increased the Homer puncta number in both neuron cultures. The lowest homer puncta number was observed in Adgrv1/del7TM astrocyte-neuron co-cultures. **c** Quantification of inhibitory post-synaptic puncta using Gephyrin staining showed significantly more puncta in Adgrv1/del7TM neuron cultures compared to WT neuron cultures. While astrocyte co-cultures increased the numbers of inhibitory synaptic puncta in WT neuron co-cultures, no differences observed in Adgrv1/del7TM neuron co-cultures. **d** Excitatory post-synaptic puncta size did not change in WT neuron co-cultures with WT or Adgrv1/del7TM astrocytes. Adgrv1/del7TM neurons showed significantly smaller size of Homer puncta. Smaller size observed in Adgrv1/del7TM neurons were increased by co-culturing with WT or Adgrv1/del7TM astrocytes. **e** Similar to excitatory synapses quantification, there was a significant decrease in inhibitory synaptic puncta in Adgrv1/del7TM neurons. Highest puncta sizes were observed in neurons with WT astrocyte co-cultures. Scale bar: 10 and 20 μm. 15–16 cells in total for *n* = 3 experiments were used, and statistical evaluation was performed using Student´s t-test analysis: *p ≤ 0.05, **p ≤ 0.01, ***p ≤ 0.001

Astrocytes promote synapse formation in a variety of ways [69]. To determine whether Adgrv1 in astrocytes plays a role in synaptogenesis in neurons, we isolated primary neurons and astrocytes derived from the hippocampus of WT or Adgrv1/del7TM mice and cultured primary neurons alone or together with primary astrocytes, respectively. In DIV14 cultures, we immunostained neurons for homer, a post synaptic density (PSD) marker for excitatory synapses, gephyrin as a PSD marker for inhibitory synapses, and the neurite marker Map2. This triple labelling allowed us to determine the number of PSD per 100 μm neurite length ~ (synaptic density) and sizes of the PSD puncta (~ synaptic strength) [70], and to distinguish between the excitatory and inhibitory synapses in neurites (Fig. 10a).

In neuron cultures of WT or Adgrv1/del7TM mice, there were no differences in the density of excitatory PSDs (homer puncta) on neurites while the density of inhibitory PSDs (gephyrin puncta) was slightly increased (Fig. 10b, c). In co-cultures of WT neurons with astrocytes from either WT and Adgrv1/del7TM mice, the density of synaptic puncta was significantly increased in both excitatory and inhibitory synapses (Fig. 10b, c) compared to neuron cultures. Co-culturing of Adgrv1/del7TM neurons with both WT and Adgrv1/del7TM astrocytes, respectively, did not affect the inhibitory PSD puncta density on neurites (Fig. 10c). However, excitatory PSD puncta density on Adgrv1/del7TM neurites increased to the level of WT neurites in co-cultures with WT astrocytes but decreased in co-cultures with Adgrv1/del7TM astrocytes (Fig. 10b). Our data suggests that Adgrv1 in astrocytes controls the frequency of excitatory synapses on neurites but has only a modest effect on the frequency of inhibitory synapses. This may lead to an imbalance of excitatory-inhibitory (E/I) synapses which has been previously correlated with neurological diseases such as epilepsy [71].

Next, we measured and quantified the size of the synaptic puncta stained for homer and gephyrin, a feature previously shown to be related with synaptic strength [70] (Fig. 10d, e). In WT neurons the puncta size for both excitatory and inhibitory PSDs was not affected by co-cultured WT and Adgrv1/del7TM astrocytes. However, in neuron cultures, the sizes of the excitatory and inhibitory PSDs were significantly decreased (*p*-value 1.29987e^− 19^ and 1.79652e^− 18^, respectively) in Adgrv1/del7TM compared to WT control mice. This was compensated in co-cultures with Adgrv1/del7TM or WT astrocytes but were more prominent by WT astrocytes (Fig. 10d, e).

In summary, our data demonstrates that Adgrv1 expression in neurons significantly affects the size of PSDs which correlates with a reduced synaptic strength in Adgrv1-deficient neurons. Furthermore, our data show that the synaptic strength in Adgrv1-deficient neurites increases by the interaction with astrocytes and that Adgrv1 in astrocytes can potentiate this effect.

## Discussion

The high expression of ADGRV1 in astrocytes is indicative of its major importance for astrocyte functions which was confirmed by our omics data: Using affinity proteomics, we identified nearly 300 proteins enriched in the proteome of human and mouse astrocytes [57] as potential interacting proteins of ADGRV1. In addition, transcriptome analyses of mouse hippocampus and human cells deficient for ADGRV1 revealed that numerous genes encoding for proteins with important functions in astrocytes were differentially expressed.

Furthermore, our omics data analysis suggests that ADGRV1 is associated with signaling pathways that are highly specific for astrocytes and essential for their proper function. One of the major functions of astrocytes in the brain is the rapid clearance of excitatory neurotransmitter glutamate from the synaptic cleft after its release from the pre-synapse to prevent glutamate neurotoxicity [72, 73]. Strikingly, we identified the glutamate transporters EAAT3 and EAAT1 (Glast) [66, 74] as potential interacting partners of ADGRV1 and experimentally validated the interaction of ADGRV1 with Glast homomultimers which were previously described [75, 76]. Although, we did not find altered protein expression of glutamate transporters in Adgrv1-deficient cells, the interaction of ADGRV1 with these core proteins of the glutamate uptake machinery suggests a role in the regulation of glutamate uptake processes in astrocytes. This is supported by our data from in vitro glutamate uptake assays and by live cell imaging of glutamate reporter, which consistently demonstrated reduced glutamate uptake in primary astrocytes deficient for Adgrv1 confirming that ADGRV1 participates in glutamate uptake in astrocytes. Interestingly, glutamate uptake by astrocytes could be triggered by the synthetic activator mimicking the tethered agonist peptide [3]. This stimulation suggests that glutamate uptake is controlled by the activation of the CTF receptor part of ADGRV1 and is not due to its cell adhesion function. Failure in glutamate uptake by astrocytes and thereby of the clearance of glutamate form the extracellular environment can lead to glutamate toxification in the brain and lead to glia and neuron cell death [77]. The toxicity of excess glutamate in the extracellular *milieu* is probably the cause for the astrocyte depletion we found in the hippocampus Adgrv1/del7TM mice.

After uptake by astrocytes, glutamate can be converted into the non-toxic essential amino acid glutamine catalyzed by GS as part of the glutamate-glutamine cycle between astrocytes and neurons [67]. Alternatively, glutamate can be also metabolized in astrocytes by the TCA cycle which operates inside the mitochondria [31]. A balanced interaction of the two pathways is vital for glutamate homeostasis in astrocytes and globally in the brain [78]. The identification of potential interacting proteins of ADGRV1 and DEGs in Adgrv1/del7TM mice related to both pathways collectively support a vital role of ADGRV1 in glutamate homeostasis. This role is further supported by the dysregulation of two key enzymes of the two pathways in Adgrv1-deficient astrocytes, namely an increase of the activity of the glutamate dehydrogenase (GDH) in the TCA and the glutamate synthetase (GS) a pivotal enzyme in the glutamate-glutamine cycle.

Interestingly, the increase of the activity of the TCA enzyme GDH is accompanied in Adgrv1-deficient astrocytes by a drastic reduction in the expression of the GS protein, a key enzyme in the glutamate-glutamine cycle. The downregulation of GS in ADGRV1-deficient hippocampal astrocytes is likely due to deficient glutamate uptake, as described in previous studies, leading to accumulation of toxic intracellular glutamate in astrocytes [79]. Intriguingly, a decrease of GS expression accompanied with an increase of GDH activity was previously described in patients with myoclonic absence epilepsy with sclerotic hippocampus [80–82] a clinical condition that also occurs in ADGRV1-associated epilepsy [16]. Overall, our results suggest that ADGRV1 is significantly involved in the control of glutamate homeostasis, which is imbalanced by defects in ADGRV1, which is proven to be disease relevant [83].

In the brain, ADGRV1 is strongly expressed in neurons, but highest in astrocytes [23]. Here, we observed that deficiency of ADGRV1 leads to decreased abundance and altered morphology of astrocytes in the CA1 region of the hippocampus, most likely due to an imbalanced glutamate homeostasis. Corresponding changes in astrocyte morphology and number have been observed in diseases in which astrocytes are known to be involved in pathogenesis such as acute brain trauma and chronic neuropathies, e.g.., Alzheimer’s disease, but also in the development of epilepsy [84–86]. The increase in branching or convex hull area of astrocytes is thought to be a consequence of the reduced number of cells in the diseased brain due to their exploratory attempts to contact the remaining neurons.

In the present study, we demonstrate that ADGRV1 is not only important for the functions of astrocytes but also of neurons. In developing neurons, the lack of ADGRV1 compromises neurite synaptogenesis. This is consistent with previously found interactions of ADGRV1 with numerous components of the pre- and post-synapse and with molecules important for synaptic signaling and neurotransmitter vesicle cycle [56]. The previously identified interactions of ADGRV1 with other synaptic molecules, such as latrophillin 2 (ADGRL2), another aGPCR [87], might be vital for synaptic function and synaptogenesis. This hypothesis supports our finding that expression of Adgrv1 in astrocytes is crucial for the size of excitatory and inhibitory synapses and thereby for synaptic strength [88, 89]. Interestingly, the presence of Adgrv1 in astrocytes influences only the abundance of excitatory synapses on neurites, but not of inhibitory synapses. This possibly leads to an imbalance of excitatory-inhibitory (E/I) synapses previously correlated with neurological diseases such as epilepsy [71].

In addition, we found that neurite branching was significantly reduced in ADGRV1-deficient neurons. This reduction is consistent with the identification of DEGs in patient cells with mutations in *ADGRV1* that encode proteins related to neuronal development and neuronal migration as well as the development of brain regions. These results demonstrate a possible role of ADGRV1 in neurite outgrowth during neurogenesis previously proposed [3].

The molecular crosstalk between astrocytes and neurons is crucial for the correct development, function, and maintenance of the proper health of the CNS [28, 90]. Here, we studied the contribution of ADGRV1 in astrocyte-neuron communication in co-cultures of primary cells from the mouse hippocampus. We found that developing primary WT neurons were supported in their development by the presence of WT astrocytes but not by Adgrv1-deficient astrocytes. In addition, the defective development of Adgrv1/del7TM primary neurons was rescued by the presence of WT primary astrocytes but was potentiated by Adgrv1-deficient astrocytes. Our findings suggest that the bidirectional interaction of ADGRV1 in astrocyte-neuron communication is supportive for the correct development of neurons.

Pathogenic variants of *ADGRV1* cause quite distinct diseases such as human Usher syndrome type 2 leading to hereditary deaf blindness [9] and various forms of epilepsy in human and rodents [13–17, 23]. It can be assumed that the loss of the fibrous membrane links formed by the extracellular domain of ADGRV1 cause crucial defects in the sensory cells of the inner ear and eye leading to USH2C [5, 91]. It is possible, but there is currently no evidence that ADGRV1 dysfunction in astrocytes may also contribute to USH disease in the eye and inner ear.

The data presented in this paper provides first insights into the molecular and cellular basis underlying epilepsy associated with mutations in *ADGRV1*. Present omics data identified molecules as potential interaction partners of ADGRV1 related to glutamate homeostasis (see above) have been previously also associated with epilepsy [92–96]. The absence of ADGRV1 from these protein complexes may also result in pathways altered in epilepsy. This is consistent with our finding that DEGs found in the hippocampus of Adgrv1/delTM7 mice and in patient-derived cells with ADGRV1 deficiency have previously been associated with epilepsy. Interestingly, the morphological and physiological alterations in astrocytes were pronounced especially in the CA1 of the hippocampus, a region which is vulnerable to glutamate toxicity [97], where recently astrocyte loss was related to early epileptogenesis [84]. Cumulatively, our data supports the hypothesis that the molecular origin of ADGRV1-associated epilepsy lies in the dysfunction of astrocytes in the hippocampus due to impaired glutamate homeostasis and its consequences caused by ADGRV1 deficits.

## Conclusion

We show here that ADGRV1 is crucial for the morphology and physiology of hippocampal astrocytes. ADGRV1 deficiency imbalances glutamate homeostasis possibly leading to glutamate toxification in the cell and neuronal tissue. The resulting consequences presumably lead to impaired neuronal development and to impaired communication between astrocytes and neurons in the brain. These molecular dysfunctions of ADGRV1-deficient astrocytes provide first clues to understanding the pathomechanisms in epilepsy associated with mutations in *ADGRV1* and will be essential in the development of future therapies. Future studies aimed at the physiology of receptor complexes in astrocytes and neurons related to ADGRV1 and the pathways downstream to activated ADGRV1 could assist in defining the precise mechanisms of ADGRV1 functions in the CNS in health and disease. This should also provide further targets for therapies and offer prospects for the future treatment and cure of ADGRV1-related epilepsy.

## Supplementary Information

Below is the link to the electronic supplementary material.

Supplementary Material 1.

Supplementary Material 2.

Supplementary Material 3.

Supplementary Material 4.

## Data Availability

Full Western blots presented in the study are included in the article/Supplementary Material. TAP data have been deposited to the ProteomeXchange Consortium via the PRIDE partner repository with the dataset identifier PXD042629. Codes for the RNA-sequencing analysis can be found at https://github.com/LabWolfrum.

## Abbreviations

ABR: Auditory brainstem response
aGPCR: Adhesion G protein-coupled receptor
Calxß: Calx-beta
CA: Cornu ammonis regions of the hippocampus (CA1-3)
CNS: Central nervous system
CTF: C-terminal fragment
DAPI: 4,6-Diamidino-2-Phenylindol
DIV: Day in vitro
DEG: Differentially expressed gene
EAAT: Excitatory amino acid transporter
ECD: Extracellular domain
ECL: Extracellular loops
EAR/EPTP: Epilepsy-associated/Epimptin like
ER: Endoplasmic reticulum
ERG: Electroretinogram
GAIN: GPCR autoproteolysis-inducing (domain)
GS: Glutamine synthetase
GSEA: Gene set enrichment analysis
Gfap: Glial fibrillary acidic protein
Glast: Glutamate aspartate transporter
GDH: Glutamate dehydrogenase
GPS: GPCR proteolysis site
GPCR: G protein-coupled receptor
GTP: Guanosine triphosphate
h: Hour
ICD: Intracellular domain
kDa: Kilo dalton
Lamg/PTX: pentaxin/laminin G-like domain
min: Minute
MAM: Mitochondria-associated ER membrane
ms: Millisecond
NTF: N-terminal fragment
PN: Postnatal day
PBM: PDZ-Binding motif
PDZ: Domain, named after PDZ containing proteins PSD95, DLG-1, and ZO-1
PSD: Post synaptic density
PTX: EAR/EPTP
RFP: Red fluorescent protein
Sox9: SRY-box transcription factor
TAP: Tandem affinity purification
TCA: Tricarboxylic acid cycle
TM: Transmembrane domain
USH: Usher syndrome
USH2C: Usher syndrome type 2 C
VLGR1: Very large G protein coupled receptor
WT: Wild type

## Declarations

### Dual publication

All results/data/figures in this publication have not been published elsewhere, nor are they under consideration by another publisher.

### Third party material

There is no material from a third party included. All of the material is owned by the authors.
