# Supplementary material for: The adhesion GPCR ADGRV1 controls glutamate homeostasis in hippocampal astrocytes supporting neurons
Source: Acta Neuropathol Commun. 2026 Apr 19;14:98. doi: 10.1186/s40478-026-02282-2 (PMC13101372; doi:10.1186/s40478-026-02282-2)
Supplement: Supplementary file 4 — Supplementary Material 4. [file 40478_2026_2282_MOESM4_ESM.docx]

**Supplemental information**

**The adhesion GPCR ADGRV1 controls glutamate homeostasis in hippocampal astrocytes supporting neurons**

Baran E. Güler^1^, Mark Zorin^1,2^, Joshua Linnert^1^, Kerstin Nagel-Wolfrum^1,2^, Uwe Wolfrum^1^*

^1^Institute of Molecular Physiology, Molecular Cell Biology, ^2^Institute of Developmental Biology and Neurobiology, Johannes Gutenberg University Mainz, 55099 Mainz, Germany.

^*^Corresponding author: Institute of Molecular Physiology, Molecular Cell Biology, Johannes Gutenberg University Mainz, Hanns-Dieter-Hüsch-Weg 17, 55128 Mainz, Germany; e-mail: [wolfrum@uni-mainz.de](mailto:wolfrum@uni-mainz.de)

Figures S1-S5

Supplementary Tables S1-S3

**Supplementary Figures and Legends**


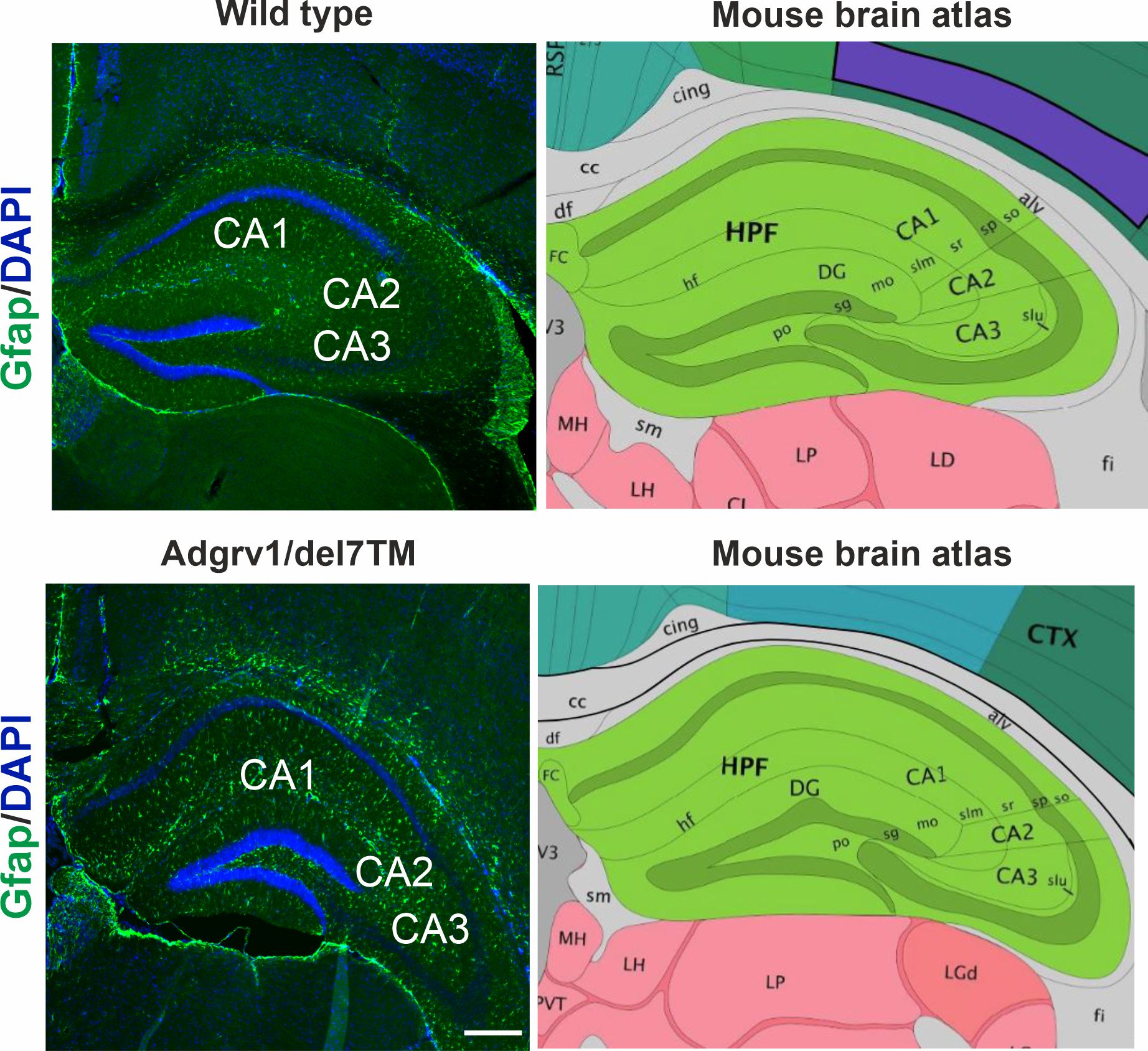


**Figure S1.** **Reference coronal brain sections from mouse brain atlas for the identification of hippocampus subregions.**

The subregions of the mouse hippocampus sections were identified using the reference atlas during the image analysis. Scale bar: 25 µm.


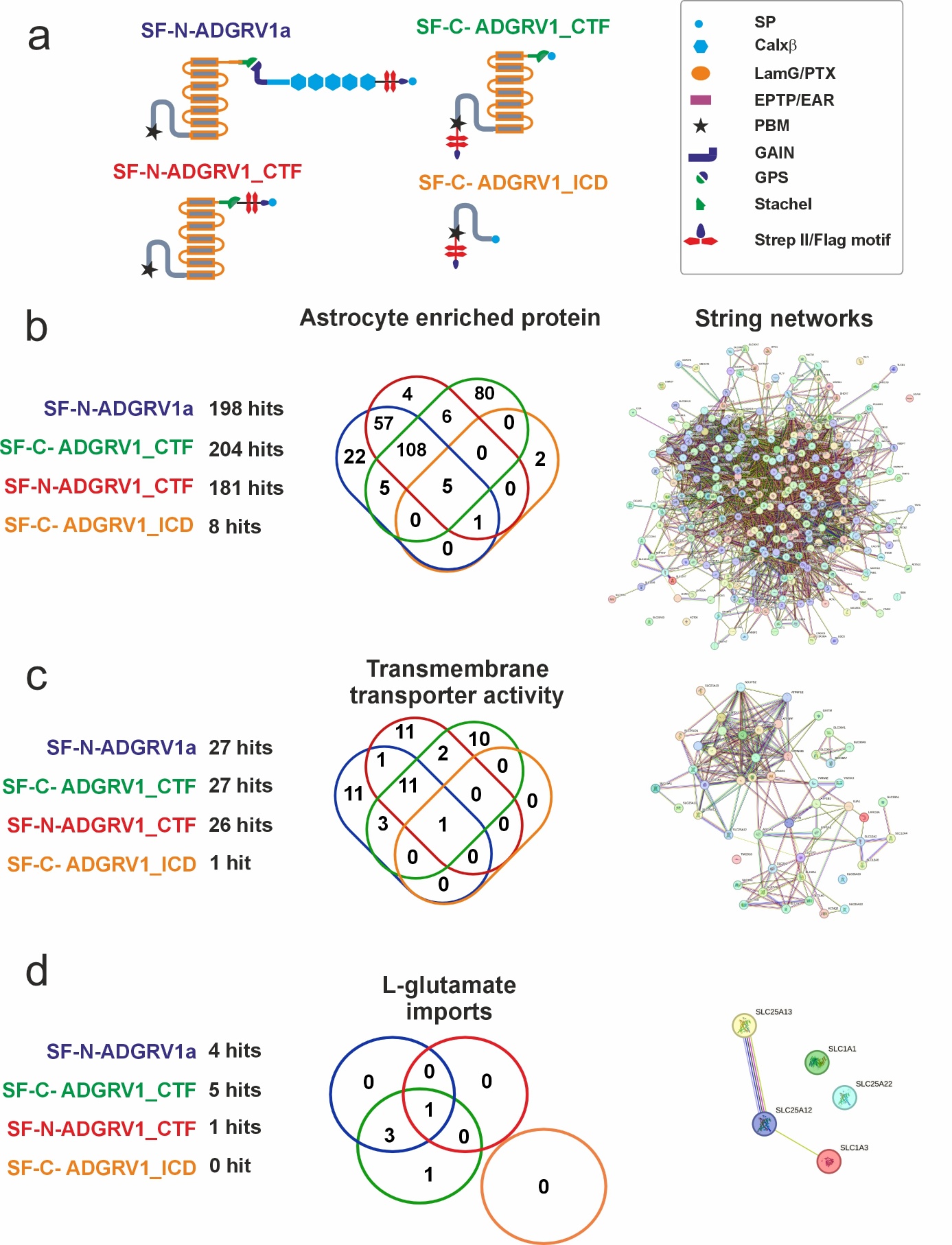


**Figure S2. TAP analysis reveals a complex protein network related to ADGRV1 protein.**

Different Strep II/FLAG (SF)-tagged ADGRV1 constructs used as a prey in Tanden affinity purification (TAP) to revealing potential interaction partners of ADGRV1 using HEK293T cells. ADGRV1 constructs were tagged from C or N terminals to eliminate false binding partners and changes in receptor structure. (**b**) GO term analysis revealed astrocyte enriched protein in ADGRV1 TAP analysis. The Venn-diagram shows a high overlap in astrocyte enriched proteins in 4 different ADGRV1 constructs, and string network analysis shows interactions. (**c**) Transmembrane transporter activity related proteins highly overlapped in SF-N-ADGRV1a, SF-N-ADGRV1_CTF and SF-C-ADGRV1 preys. (**d**) L-glutamate import related proteins have enriched in SF-N-ADGRV1a and SF-C-ADGRV1 preys.


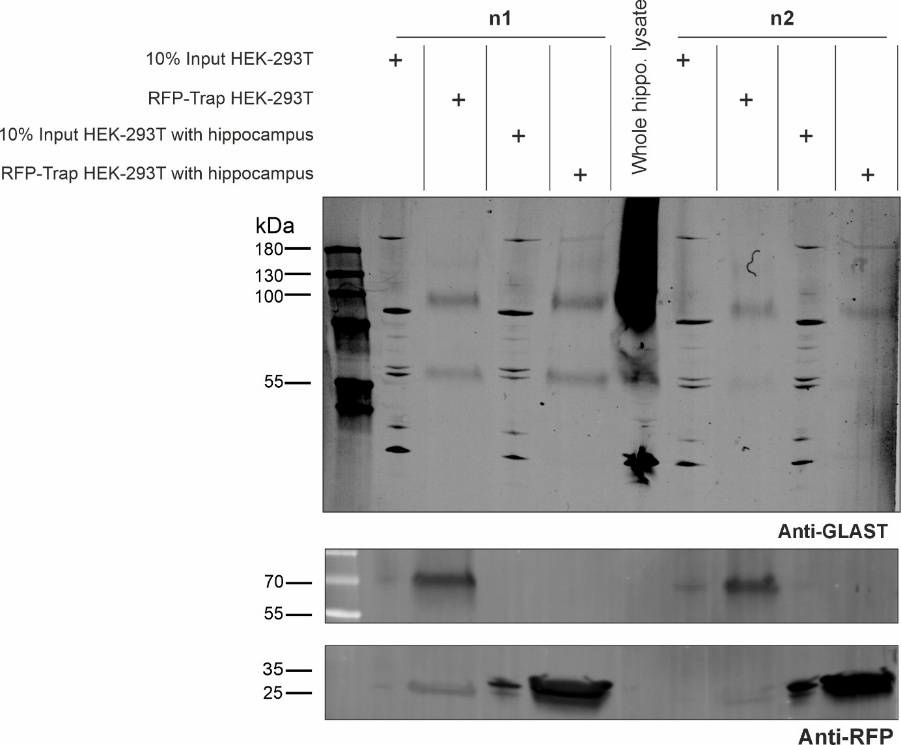


**Figure S3. Repetition of the pull-down experiment on the Glast glutamate transporter in the hippocampus by RFP-labelled ADGRV1.**

RFP-TRAP pull-down of Glast by RFP-ADGRV1_CTF. RFP-Trap pull-down assays were performed using lysates from HEK-293T cells transfected with RFP-ADGRV1-CTF or RFP-only (control) and whole hippocampal lysate. Immunoblotting with anti-GLAST antibodies shows that Glast co-precipitates with RFP-ADGRV1-CTF, but not with the RFP-only control. Top Panel (Anti-GLAST): Endogenous GLAST expression was detected in the "10% Input" and "Whole hippo. lysate" lanes. GLAST appears as monomeric bands (~60 kDa) and high-molecular-weight multimeric complexes (above 180 kDa). Notably, GLAST multimers were successfully co-immunoprecipitated in the RFP-Trap HEK-293T with hippocampal lysates, indicating a biochemical interaction between GLAST and the ADGRV1 C-terminal fragment.


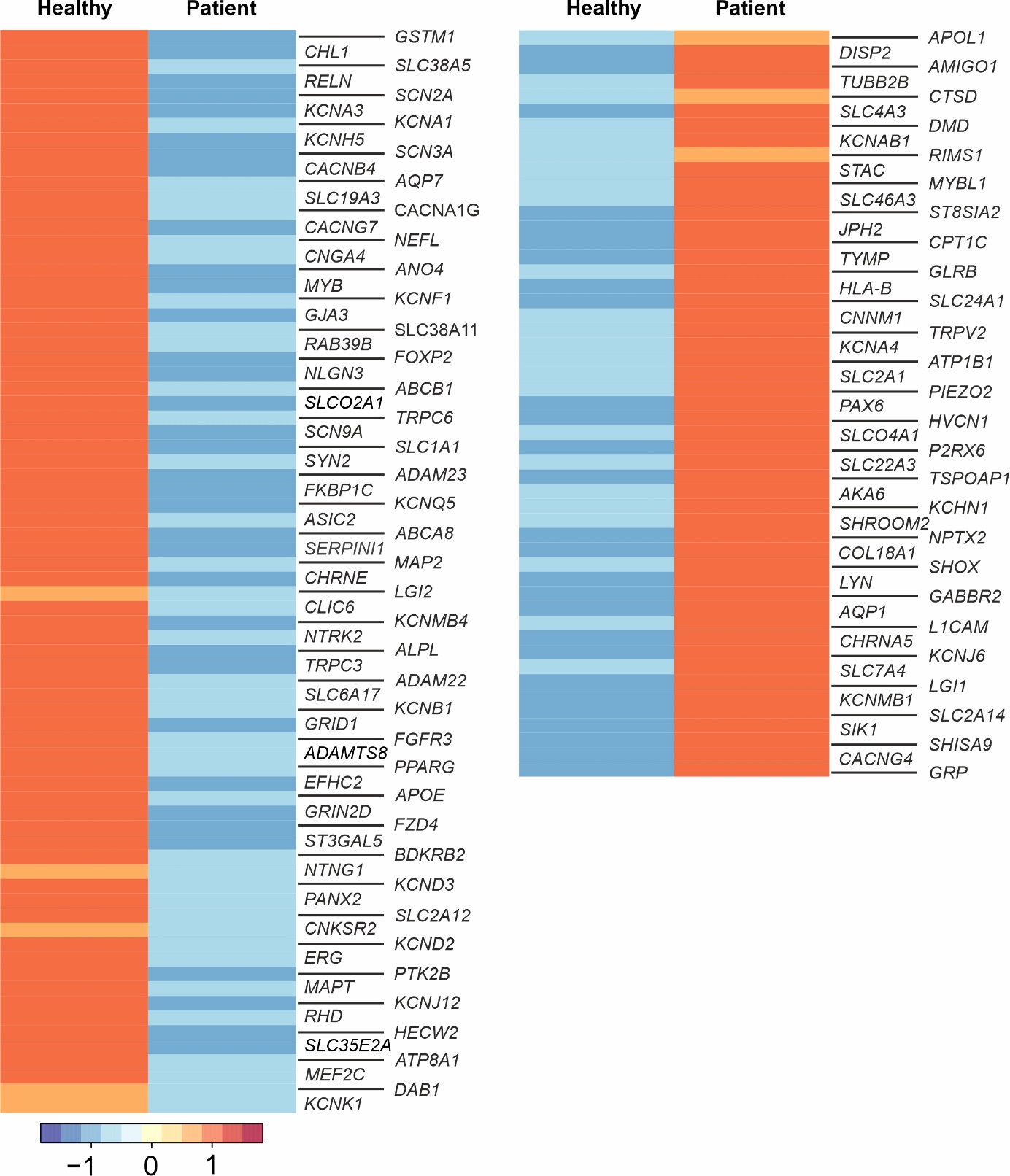


**Figure S4. Differential expressed genes (DEGs) related to glutamate homeostasis and epilepsy in *ADGRV1*^Arg2959*^patient-derived fibroblasts compared to fibroblasts from a healthy individuum.**

The average expression profiles of 3 replicates of healthy individuum and patient fibroblasts is shown. Blue color shows downregulated genes and red color shows upregulated genes in *ADGRV1*^Arg2959*^ patibluederived dermal fibroblasts compared to healthy individuals. 74 genes are up, and 51 genes are downregulated in *ADGRV1*^Arg2959*^ patient-derived fibroblasts.


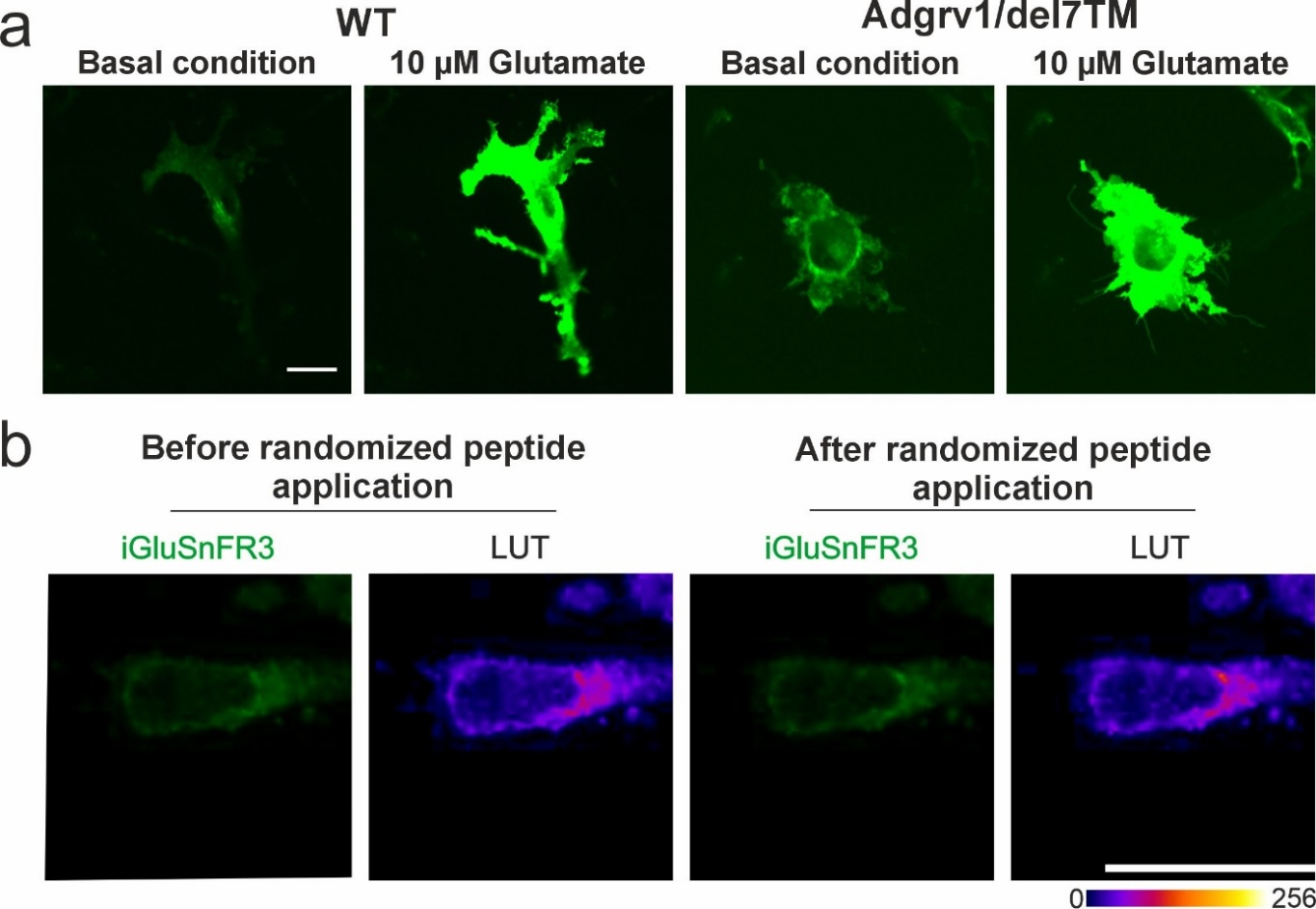


**Figure S5. Controls to live-cell imaging of glutamate sensing by pAAV.GFAP.iGluSnFR3.v857.GPI reporter in WT and Adgrv1/del7TM astrocytes**.

(**a**) Functional validation of iGluSnFR3 sensor. Time-lapse imaging of primary astrocytes expressing pAAV.GFAP.iGluSnFR3.v857.GPI (green) was performed at 700 ms intervals over a 300-second duration. Representative images show fluorescence under Basal conditions and following the exogenous application of 10 µM Glutamate (applied at 98 sec) in both WT and Adgrv1/del7TM cells. The robust increase in green fluorescence confirms the sensitivity and responsiveness of the iGluSnFR3 sensor in both genotypes (response at 100 sec) Scale bar: 20 µm. (**b**) Specificity control using randomized peptide. Representative images of an astrocyte expressing iGluSnFR3 before and after the application of a randomized control peptide (scrambled sequence of the 11 amino acids of the Adgrv1 Stachel peptide). Left panels show raw iGluSnFR3 fluorescence; right panels show the corresponding Look-Up Table (LUT) heatmaps to visualize intensity changes. The lack of significant fluorescence increase following application demonstrates that the glutamate-sensing response is specific to the active Stachel sequence and not a non-specific effect of peptide addition. Scale bar: 25 µm; LUT scale: 0–256.
